# Anti-IgD nanobodies as novel tools for studying human IgD biology

**DOI:** 10.1038/s41598-025-09118-4

**Published:** 2025-07-08

**Authors:** Susan K. Vester, Rebecca L. Beavil, Alexander Alexandrovich, Hannah J. Gould, Andrew J. Beavil, Brian J. Sutton, James M. McDonnell

**Affiliations:** 1 Randall Centre for Cell and Molecular Biophysics, King’s College London, New Hunt’s House, London, SE1 1UL UK; 2 Present Address: 272BIO, Kent Science Park, Building 400, The Ventures Building, Sittingbourne, ME9 8AG UK

**Keywords:** Antibody, Bispecific, Fc, Immunoglobulin D (IgD), Nanobody, Surface plasmon resonance, Biochemistry, Biophysics, Immunology

## Abstract

IgD is the least well characterized of mammalian antibody isotypes and its biology remains poorly understood. Nanobodies are a useful and versatile tool for research and diagnostics, including for protein purification, capture and detection applications. Here we report the characterization of four anti-human IgD nanobodies, specific to the Fc region of IgD. The four nanobodies bound to human IgD with low nanomolar affinity, showing different binding kinetics and interaction stoichiometries, as well as recognizing four distinct epitopes. We found one of these anti-IgD nanobodies, aδNb408, to be an excellent purification tool for IgD, with efficient capture and elution at pH 3.5. To aid the analysis of IgD interactions using surface plasmon resonance, we identified two nanobodies that provided good capture of IgD. Assembling bispecific and bivalent nanobody pairs increased the avidity of the anti-IgD nanobodies, with the bispecific nanobody pair aδNb408-aδNb107 showing robust detection of IgD on Namalwa B cells. The anti-IgD nanobody tools described here can be used for diverse applications that have the potential to further our understanding of IgD biology.

**Supplementary Information:**

The online version contains supplementary material available at 10.1038/s41598-025-09118-4.

## Introduction

Antibodies play an important role in the immune system and are crucial for protection against pathogens^1^. Out of the five human antibody classes (IgA, IgD, IgE, IgG and IgM), IgD remains the least well characterized isotype. In transmembrane form, IgD acts as B cell antigen receptor, and it has roles in peripheral tolerance via its involvement in B cell anergy^2^. The functions of secreted IgD, first discovered in 1965^3^, are less well understood, although considerable advances have been made in recent years. With low abundance in the serum of healthy individuals and present in the upper aerodigestive mucosa^4^, secreted IgD has been implicated in mucosal immunity, involved in interactions with the respiratory microbiota^2^. While IgD has recently been shown to bind to basophils via galectin-9 and CD44^5^, the cellular receptors of secreted IgD remain poorly understood. Structurally, human IgD follows the architecture of a typical antibody, comprised of two identical Fab regions and an Fc region, but separated by a long hinge region, giving IgD a T-shaped structure^6^. The Fc region of human IgD (IgD-Fc) comprises two Cδ2 and two Cδ3 domains. Recent studies from our group have determined high-resolution structures of IgD Fab^7^ and IgD-Fc^8^, elucidating both unique and common properties of the IgD molecule compared with the other antibody classes.

Aside from a small number of commercially available antibodies, few tools are available to study IgD. Previous purification methods for human IgD have included indirect lectin-based approaches that rely on glycosylation that is not specific to IgD, such as ricinus agglutinin^9^ or jackfruit jacalin^10^. For recombinant IgD, generic approaches for purification using light chain specificity, or tailored approaches using antigen specificity can work well. The superantigen MID, an IgD-binding protein from *Moraxella catarrhalis*, binds to the Fab region of human IgD^11^, and a two-step purification procedure of IgD using MID has been described^12^. However, having specific and straightforward tools for purification of any human IgD molecule and for other applications would be advantageous.

Nanobodies (Nbs), also known as single-domain antibodies or V_H_H, are derived from camelid heavy chain-only antibodies^13^. Their antigenic recognition potential is contained within a single domain, mostly formed by three complementarity-determining regions (CDRs), and due to their small size and stability they lend themselves to easy expression in bacteria^14^. Nbs have become versatile research tools, including for imaging applications, in protein purification, as capture and detection tools and as crystallization chaperones^15^. While many Nbs bind to their target with high affinity, monovalent Nbs do not show the same avidity effects displayed by other antibody formats.

Here we introduce four Nbs, anti-IgD Nb 107 (aδNb107), anti-IgD Nb 367 (aδNb367), anti-IgD Nb 408 (aδNb408) and anti-IgD Nb 571 (aδNb571), which bind to the Fc region of human IgD and can be used as tools for purification, surface plasmon resonance (SPR) capture and flow cytometric detection of IgD.

## Results

### Characterization of four anti-IgD Nbs

We recently described the generation of Nbs against human IgD, with an anti-idiotype Nb initially reported^16^. Here we characterize four different anti-IgD Nbs, that specifically bind to the Fc region of IgD. The Nbs were derived from immune libraries of an alpaca and a llama immunized with human anti-Phl p 7 IgD 102.1F10 (HAPPID1). aδNb107 emerged after two rounds of panning on full-length IgD, aδNb367 emerged after two rounds of panning on IgD-Fc, and aδNb408 and aδNb571 emerged after three rounds of panning on IgD-Fc. The four Nbs were from different B cell lineages based on CDR3 family (Supplementary Fig. S1).

As IgD-Fc is a homodimer, we analyzed the stoichiometry of the interaction between each Nb and IgD-Fc by size exclusion chromatography (SEC) (Supplementary Fig. S2, Table 1). At the concentrations assayed here, aδNb107, aδNb367 and aδNb408 bound to IgD-Fc with 2:1 stoichiometry, while aδNb571 bound to IgD-Fc with 1:1 stoichiometry. One possible explanation for the absence of unbound aδNb367 and aδNb408 could be that they interact with the column matrix. While the anti-IgD Nbs described here are not synthetic, as many as 5–30% of synthetic Nbs interact with commonly used Superdex 200 resins^17^.

**Table 1:** Anti-IgD nanobody characteristics.

Nanobody	Binding affinity and kinetics	Stoichiometry Nb:IgD	Domain specificity*	Isotype specificity
aδNb107	K_D1_ = 3.2 (± 0.8) × 10^–9^ M K_D2_ = 2.2 (± 0.1) × 10^–8^ M k_on_ = 2.1 (± 0.1) × 10^5^ M^−1^ s^−1^ k_off1_ = 6.6 (± 1.8) × 10^–4^ s^−1^ k_off2_ = 4.6 (± 0.1) × 10^–3^ s^−1^	2:1		IgD
aδNb367	K_D_ = 2.4 (± 0.1) × 10^–9^ M k_on_ = 2.0 (± 0.1) × 10^5^ M^−1^ s^−1^ k_off_ = 4.7 (± 0.1) × 10^–4^ s^−1^	2:1		IgD
aδNb408	K_D1_ = 8.9 (± 1.0) × 10^–10^ M K_D2_ = 5.2 (± 0.1) × 10^–9^ M k_on_ = 8.2 (± 0.1) × 10^5^ M^−1^ s^−1^ k_off1_ = 7.4 (± 0.9) × 10^–4^ s^−1^ k_off2_ = 4.3 (± 0.1) × 10^–3^ s^−1^	2:1	Cδ2	IgD
aδNb571	K_D_ = 6.6 (± 0.6) × 10^–10^ M k_on_ = 1.8 (± 0.1) × 10^6^ M^−1^ s^−1^ k_off_ = 1.2 (± 0.1) × 10^–3^ s^−1^	1:1		IgD

*aδNb107, aδNb367 and aδNb571 may bind at the Cδ2/Cδ2 interface, at the Cδ2/Cδ3 interface or may be Cδ3 binders.

**Fig. 1:**
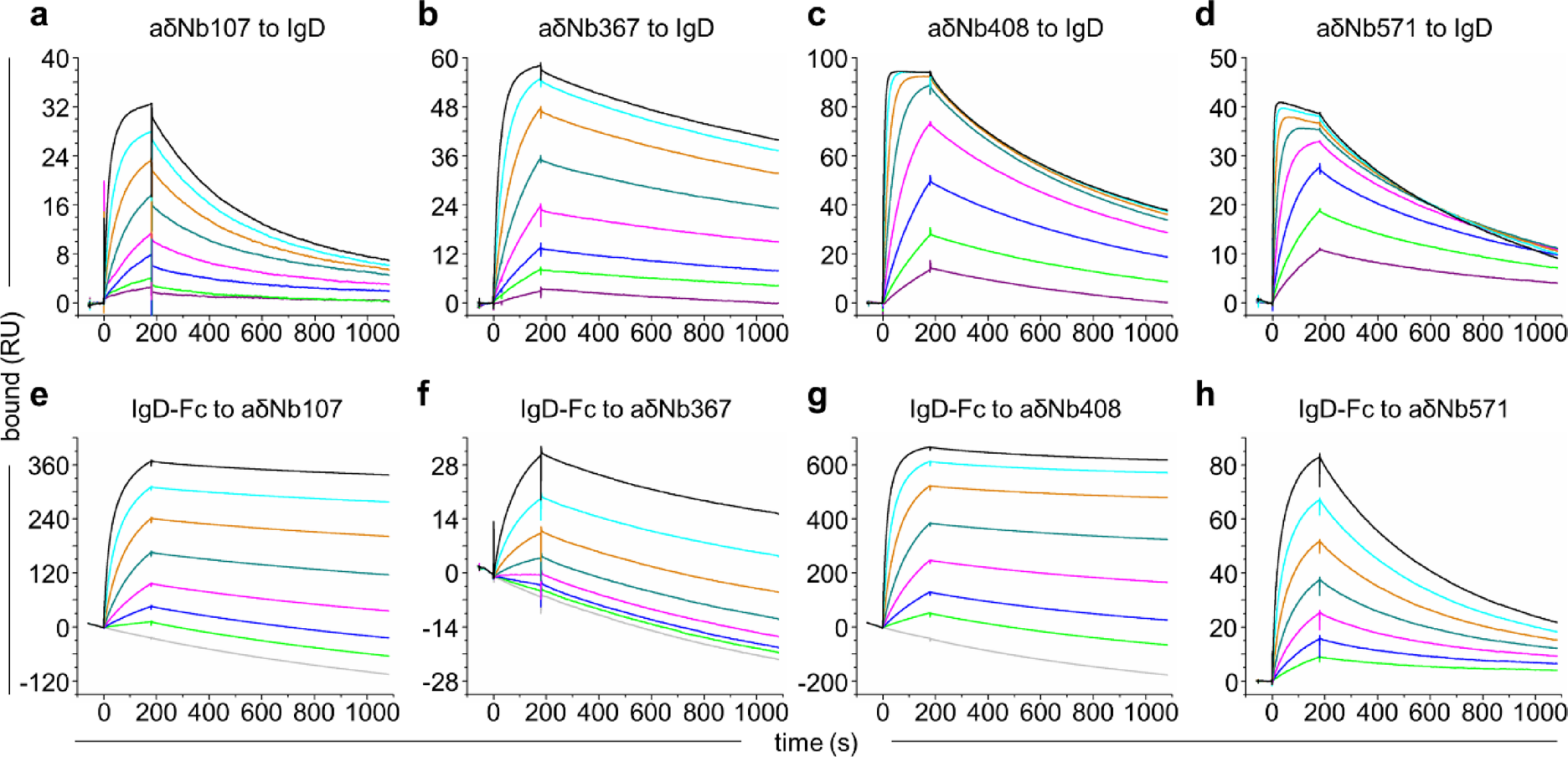
Interaction of anti-IgD Nbs with IgD. Binding of anti-IgD Nbs (**a**) aδNb107, (**b**) aδNb367, (**c**) aδNb408 and (**d**) aδNb571 to IgD captured on an anti-His chip by SPR. A two-fold dilution series of TEV-cleaved anti-IgD Nbs was flowed over His-tagged IgD, from the highest concentration 200 nM (black line) to the lowest concentration 1.6 nM (purple line). Binding of IgD-Fc to anti-IgD Nbs (**e**) aδNb107, (**f**) aδNb367, (**g**) aδNb408 and (**h**) aδNb571. The His-tagged Nbs were captured by anti-His. A two-fold dilution series of IgD-Fc was flowed over, with the highest concentration 200 nM (black line) and the lowest concentration 3 nM (green line). Double-reference subtraction was not performed for (**e**) aδNb107, (**f**) aδNb367 and (**g**) aδNb408, with the 0 nM concentration shown as a gray line. RU, resonance units.

We determined the binding affinities of aδNb107, aδNb367, aδNb408 and aδNb571 by SPR, capturing His-tagged IgD on an anti-His chip and flowing over a two-fold dilution series of Nbs (Fig. 1a–d, Table 1). Binding was to some extent biphasic; we did not report a second component in situations where this represented < 10% of the total binding. Affinities were in the low nanomolar range, between 0.6 and 22 nM. aδNb107 showed the most marked biphasicity with two binding components of a similar size; one component with a K_D_ of about 3 nM and the other ~ seven-fold weaker. This suggests that the two binding sites for aδNb107 on IgD have some asymmetry, either intrinsically or induced by the binding of the first aδNb107 molecule. aδNb367 only had a small second component and was therefore fit monophasically, with a K_D_ of about 2 nM. aδNb408 showed monophasic association and biphasic dissociation behavior, with the minor component making up around 20% (k_off2_ and K_D2_). aδNb571, a 1:1 binder, was fit monophasically and showed a fast association rate, which was responsible for its high affinity. To further assess binding using a reciprocal approach, we captured Nbs on an anti-His chip and flowed over a two-fold dilution series of IgD-Fc (Fig. 1e–h). We observed differences in the association phase when IgD-Fc was the analyte, with markedly slower association displayed. This is possibly due to unfavorable electrostatic interactions of IgD-Fc or favorable electrostatic interactions of the Nbs with the negatively charged CM5 chip^18^. In addition to this, when IgD-Fc was flowed over aδNb107, aδNb367 or aδNb408 (Fig. 1e–g), we observed artifacts in the dissociation phase, with the 0 nM reference concentration no longer being a suitable baseline. This artifact appears to be based on avidity effects. All three Nbs bind to IgD-Fc with 2:1 stoichiometry, and both binding sites are engaged when IgD-Fc is flowed over the Nbs, resulting in very slow dissociation of IgD-Fc and apparently slowing dissociation of the Nbs captured on the anti-His chip. This phenomenon was not seen for binding of IgD-Fc to aδNb571 (Fig. 1h), which as a 1:1 interaction would not be expected to show any avidity effects.

Both binding sites are engaged when IgD-Fc is captured by Nbs that show 2 Nb:1 IgD interaction stoichiometries, and we could therefore perform epitope bin analysis by SPR (Supplementary Fig. S3). Each of the four Nbs was able to bind to IgD-Fc when one of the other three Nbs was pre-bound, suggesting that aδNb107, aδNb367, aδNb408 and aδNb571 bind to different epitopes. However, when anti-His immobilized aδNb571 was used to capture IgD-Fc, each of the three other Nbs was able to induce dissociation of aδNb571. As the initial capture of aδNb571 was performed by an anti-His antibody, this could be an indirect phenomenon resulting from dissociation of the captured aδNb571 from the chip. However, it could also suggest that aδNb107, aδNb367 and aδNb408 are able to induce the dissociation of IgD-Fc from aδNb571 by either a proximal or allosteric mechanism. Binding of aδNb571 to IgD-Fc when aδNb107, aδNb367 or aδNb408 was pre-bound was not affected, suggesting different epitopes are being recognized.

Next, we wanted to investigate the domain specificity of the anti-IgD Nbs. We were able to show that aδNb408 bound to the monomeric Cδ2 domain (Supplementary Fig. S4; Table 1). aδNb107, aδNb367 and aδNb571 did not bind to monomeric Cδ2, suggesting they either bind at the Cδ2/Cδ2 interface, at the Cδ2/Cδ3 interface or are Cδ3 binders.

Finally, we tested whether the four Nbs described here specifically bind to IgD. We assessed binding to the four other human isotypes IgA, IgE, IgG and IgM at approx. 500 nM (assuming monomeric concentration, Supplementary Fig. S5). None of the anti-IgD Nbs showed cross-reactivity with other isotypes, confirming specific binding to IgD (Table 1).

### aδNb408 as an anti-IgD purification tool

**Fig. 2:**
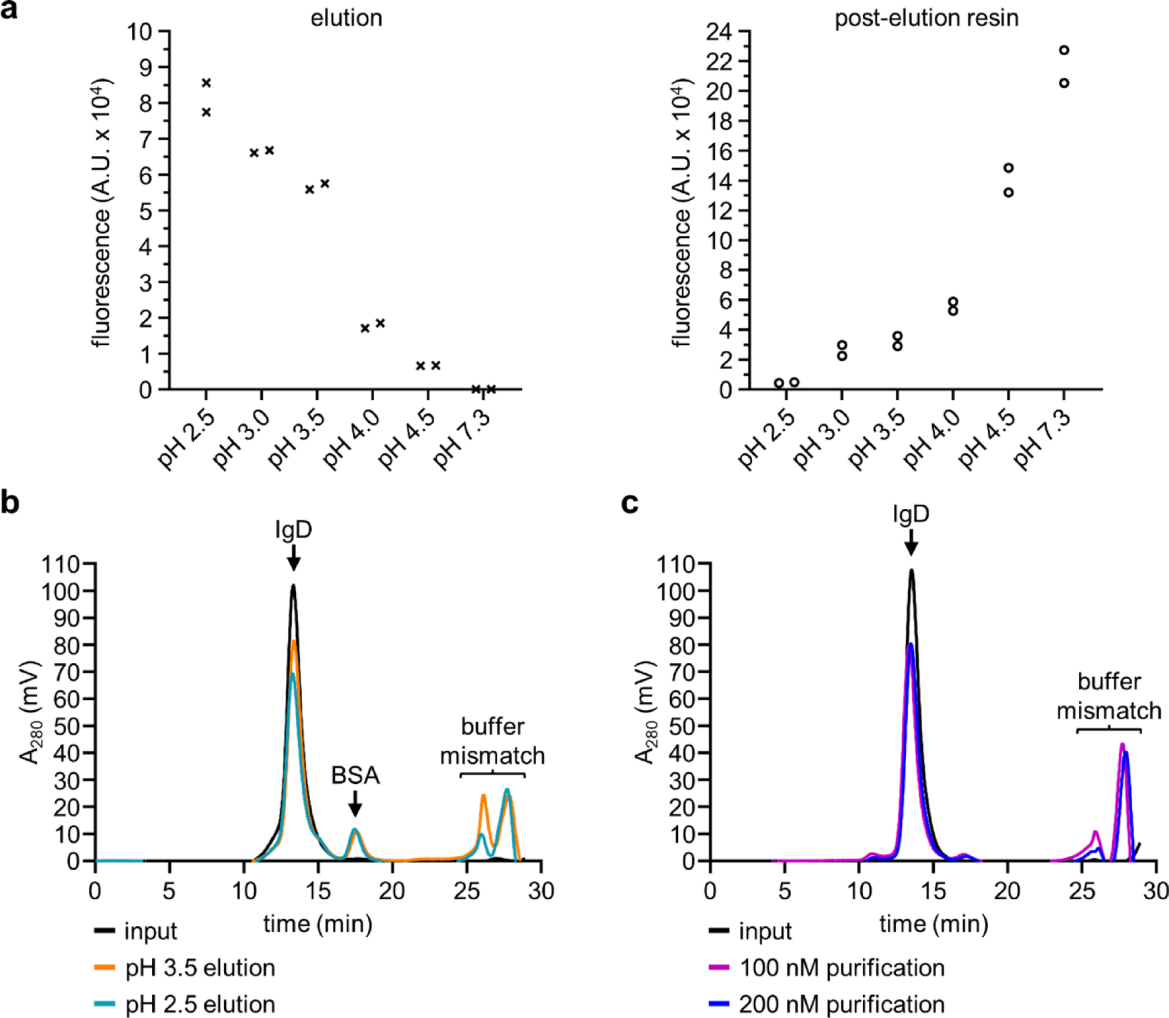
aδNb408 can be used as a purification tool. (**a**) Testing of elution conditions for the aδNb408 affinity resin. Alexa Fluor 488 (AF488)-labeled IgD-Fc was detected by measuring fluorescence of combined elution fractions (left panel) or of the aδNb408 affinity resin after elution (right panel). A.U., arbitrary units. (**b**) Size exclusion chromatogram showing IgD purification from BSA-containing buffer, with elution at pH 3.5 or pH 2.5. Input represents the maximal amount of IgD that could have been purified. The elution position of monomeric BSA is indicated. Note the buffer mismatch occurring from ~ 25 min onwards. (**c**) Size exclusion chromatogram showing purification of IgD supplemented into culture supernatant containing 10% FBS at 100 nM or 200 nM, with elution at pH 3.5. Input represents the maximal amount of IgD that could have been purified. Note the buffer mismatch occurring from ~ 25 min onwards.

Having performed a thorough characterization of the four anti-IgD Nbs described above, we wanted to explore their utility as research tools. To allow specific purification of IgD of any antigen specificity, whether κ or λ light chain, or of the IgD-Fc region only, we explored the use of anti-IgD Nbs for IgD purification. We produced an affinity resin by conjugating aδNb408 to a Sepharose matrix using NHS coupling. First, we tested feasibility of capture and elution from aδNb408. Using fluorescently labeled IgD-Fc doped into buffer containing 1% BSA at 500 nM, we tested purification of IgD-Fc with elution conditions at different pH values (Fig. 2a). Good elution was seen up to and including pH 3.5, while elution conditions above pH 4.0 had little effect upon release of IgD-Fc.

Having established successful purification of IgD-Fc, we tested purification of full-length IgD doped into buffer containing 1% BSA at 242 nM, with elution at pH 2.5 or pH 3.5 (Fig. 2b). The size exclusion chromatogram showed a single peak for IgD, with a small amount of impurity, likely BSA. The increase in A_280_ starting at around 25 min is due to buffer mismatch between the running buffer and the elution/neutralization buffer components, and these peaks are present when neutralized elution buffer only is analyzed. Purification at pH 2.5 and pH 3.5 gave nearly identical results, and we chose to use pH 3.5 elution as a gentle yet effective condition for purification. Next, we tested purification efficiency of IgD doped into complex culture supernatant at 100 nM or 200 nM (Fig. 2c), which is at the lower end of average serum IgD concentrations in healthy individuals^4^. Again, SEC showed a major single peak for IgD, with very small amounts of impurities present. No difference was seen in the purification efficiency at 100 nM or 200 nM, and these concentrations are well above the binding affinity of aδNb408. We purified full-length IgD and IgD-Fc used in the following experiments by aδNb408 affinity chromatography and found it to be fully active. The resin could be re-used multiple times over a period of 12 months without noticeable loss of performance.

### Anti-IgD Nbs as capture tools for SPR

**Fig. 3:**
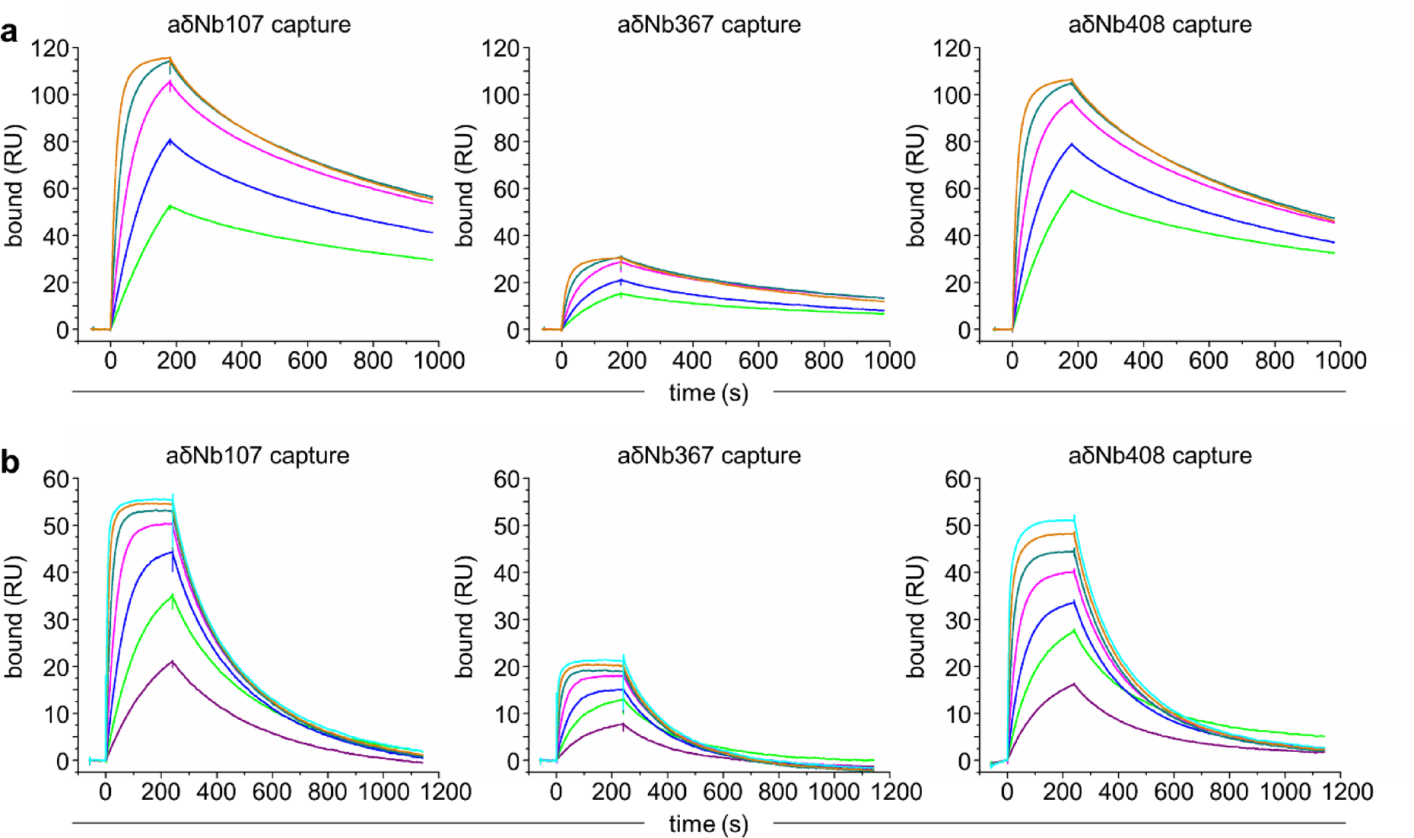
Anti-IgD Nbs as tools for SPR capture. Biotinylated aδNb107, aδNb367 and aδNb408 were immobilized onto an SA chip. (**a**) IgD-Fc was captured by anti-IgD Nbs and a two-fold dilution series of aδNb571 was flowed over, with the highest concentration 50 nM aδNb571 (orange line) and the lowest concentration 3 nM aδNb571 (green line). (**b**) Full-length IgD, HAPPID1, was captured by anti-IgD Nbs and a two-fold dilution series of the polcalcin allergen Ole e 3 was flowed over, with the highest concentration 100 nM (cyan line) and the lowest concentration 1.6 nM (purple line). RU, resonance units.

It has been our experience that IgD directly immobilized onto an SPR sensor chip does not lend itself to experiments requiring standard regeneration conditions such as multiple rounds of regeneration at low pH. Ways to circumvent this problem include capturing IgD through affinity tags (as performed here using His-tag capture for recombinant IgD) or (super)antigen capture. However, studying binding interactions between IgD, its antigens, superantigens or other binders such as a cellular receptor or co-receptor would benefit from tools able to specifically capture IgD as a ligand for SPR analysis. To this end we tested the four anti-IgD Nbs described here as capture tools. While we have observed that direct conjugation of Nbs to a CM5 sensor chip via NHS coupling can be successful, a gentler approach is to perform biotinylation of the Nbs. We biotinylated the Nbs using NHS coupling and captured them on a streptavidin (SA) chip. aδNb571 was not a reliable capture tool for IgD-Fc, as it did not withstand low pH regeneration well, leading to low levels of capture and analyte binding (Supplementary Fig. S6). aδNb107, aδNb367 and aδNb408 withstood regeneration well enough to allow for repeated capture of IgD-Fc or IgD. We tested capture of IgD-Fc and binding of a two-fold dilution series of aδNb571 (Fig. 3a). Similarly, we tested capture of IgD and binding of a two-fold dilution series of the polcalcin antigen Ole e 3 (Fig. 3b), to which the IgD used here, HAPPID1, shows cross-reactivity. Dissociation of captured IgD-Fc or IgD was very slow from all three Nbs, as expected due to avidity effects. Capture by aδNb367 was much poorer than for aδNb107 and aδNb408 (as seen previously when assessing affinities), making this a less suitable capture tool. However, both aδNb107 and aδNb408 can be used as robust capture tools for SPR experiments. The availability of aδNb408 (a confirmed Cδ2 binder) and aδNb107 (a probable Cδ3 binder) as capture tools from distinct epitope bins will facilitate further investigation of IgD interactions.

### Anti-IgD Nb pairs as IgD detection tools

**Fig. 4:**
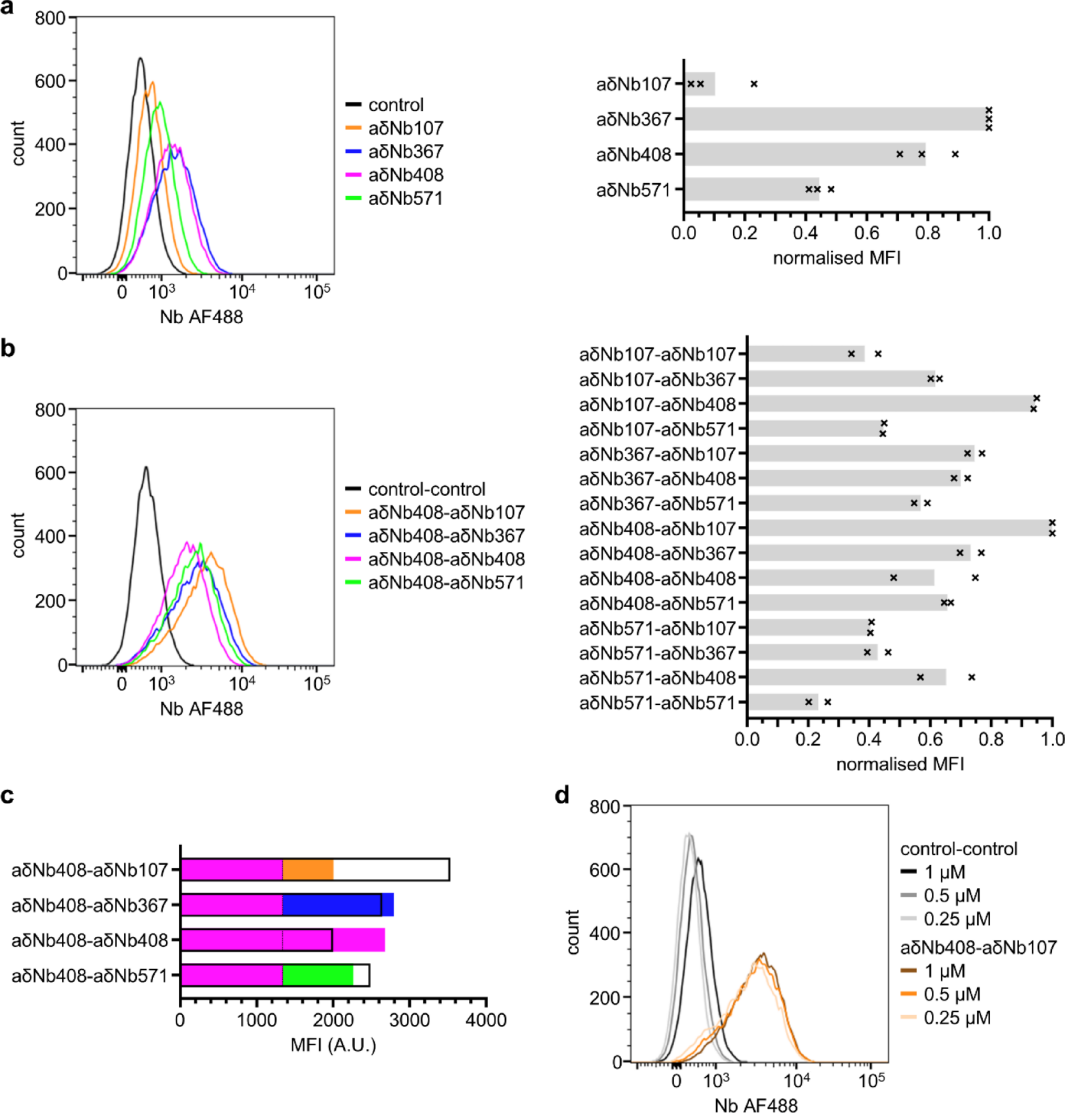
Anti-IgD Nb pairs as a tool for cell-binding. (**a**) Left: Flow cytometry data of AF488-labeled anti-IgD Nbs binding to Namalwa cells. A non-IgD binding Nb was used as a control for non-specific background binding. Right: MFI was normalized to the control and the highest binder aδNb367, with the mean plotted as a gray bar and the individual data points as black crosses (n = 3). (**b**) Left: Flow cytometry data of anti-IgD Nb pairs binding to Namalwa cells. A pair of non-IgD binding Nbs was used as a control for non-specific background binding. Right: MFI was normalized to the control and the highest binder aδNb408-aδNb107, with the mean plotted as a gray bar and the individual data points as black crosses (n = 2). (**c**) Comparison of the bivalent/bispecific aδNb408-Nb constructs (panel b, left) with their monovalent Nb components (panel a, left); these data are from the same experiment and are therefore comparable. MFI values of monovalent aδNb107 (orange, 662), monovalent aδNb367 (blue, 1452), monovalent aδNb408 (magenta, 1339) and monovalent aδNb571 (green, 921) are shown additively as filled bars, separated by a dashed line. MFI values of equivalent bivalent/bispecific Nbs are shown as empty black bars: aδNb408-aδNb107 (3533), aδNb408-aδNb367 (2641), aδNb408-aδNb408 (1998) and aδNb408-aδNb571 (2488). (**d**) Flow cytometry data of Nb pair aδNb408-aδNb107 titrated on Namalwa cells. MFI, median fluorescence intensity; AF488, Alexa Fluor 488; A.U., arbitrary units.

Next, we wanted to investigate how these four anti-IgD Nb tools fare as detection agents of IgD-positive cells in flow cytometry. We chose the IgD-expressing Namalwa B cell line for our experiments^19^. First, we reacted SpyTagged Nbs with AF488-labeled SpyCatcher003 for use as flow cytometry detection tools, with a covalent bond formed between SpyTag003 and SpyCatcher003^20^. Binding of the thus fluorescently labeled Nbs was tested to Namalwa cells (Fig. 4a, for gating see Supplementary Fig. S7). Most binding was observed for aδNb367 and aδNb408. In contrast, aδNb571 and especially aδNb107 proved to be poor cell surface IgD detection reagents. Binding was performed at 1 µM, a concentration well above the K_D_ value of these Nb interactions with soluble IgD, suggesting that differences in cell-binding are due to differences in membrane-bound IgD, and this appears to be more pronounced for possible Cδ3 binders aδNb107 and aδNb571.

To assess whether we could increase detection of IgD, we made bispecific and bivalent anti-IgD Nb constructs using the DoubleCatcher platform^21^. The DoubleCatcher platform allows SpyTagged Nbs to be easily assembled into bispecific binders, and we combined each of the four anti-IgD Nbs with one another, in both possible orders. For flow cytometry, an AF488-labeled DoubleCatcher was used. We tested binding of AF488-labeled Nb pairs to Namalwa cells as before (Fig. 4b). Bivalent aδNb367-aδNb367 was excluded from the analysis as the protein precipitated extensively during assembly. Even though monovalent aδNb107 had shown little cell-binding, fusions with aδNb107 were very successful binders. The aδNb408-aδNb107 pair showed the best cell-binding, closely followed by the aδNb107-aδNb408 construct, suggesting that the order of the two Nbs had little effect on IgD detection. Overall, conjugates containing aδNb367 or aδNb408 showed good cell-binding. When we compared the bivalent and bispecific Nb fusions with their individual monovalent Nb components (Fig. 4c), we observed that binding of some Nb pairs was similar to the sum of their components, while the aδNb408-aδNb107 fusion showed markedly better binding than the sum of its two parts. Cell-binding by aδNb408-aδNb107 was robust, with four-fold dilution of the Nb pair having little effect on detection ability (Fig. 4d). While little binding of monovalent aδNb571 and aδNb107 to Namalwa cells might have suggested that their binding site is inaccessible in the transmembrane form of IgD, improvement of binding for Nb pairs containing aδNb107 suggests that its epitope is accessible and stabilizes the interaction. In the DoubleCatcher construct we used, the two Nbs aδNb408 and aδNb107 could be at a good spacing distance to simultaneously interact with IgD. This interaction could be mediated by either intramolecular binding or intermolecular cross-linking of IgD by the Nb pair.

### Further characterization of anti-IgD Nb pairs

**Fig. 5:**
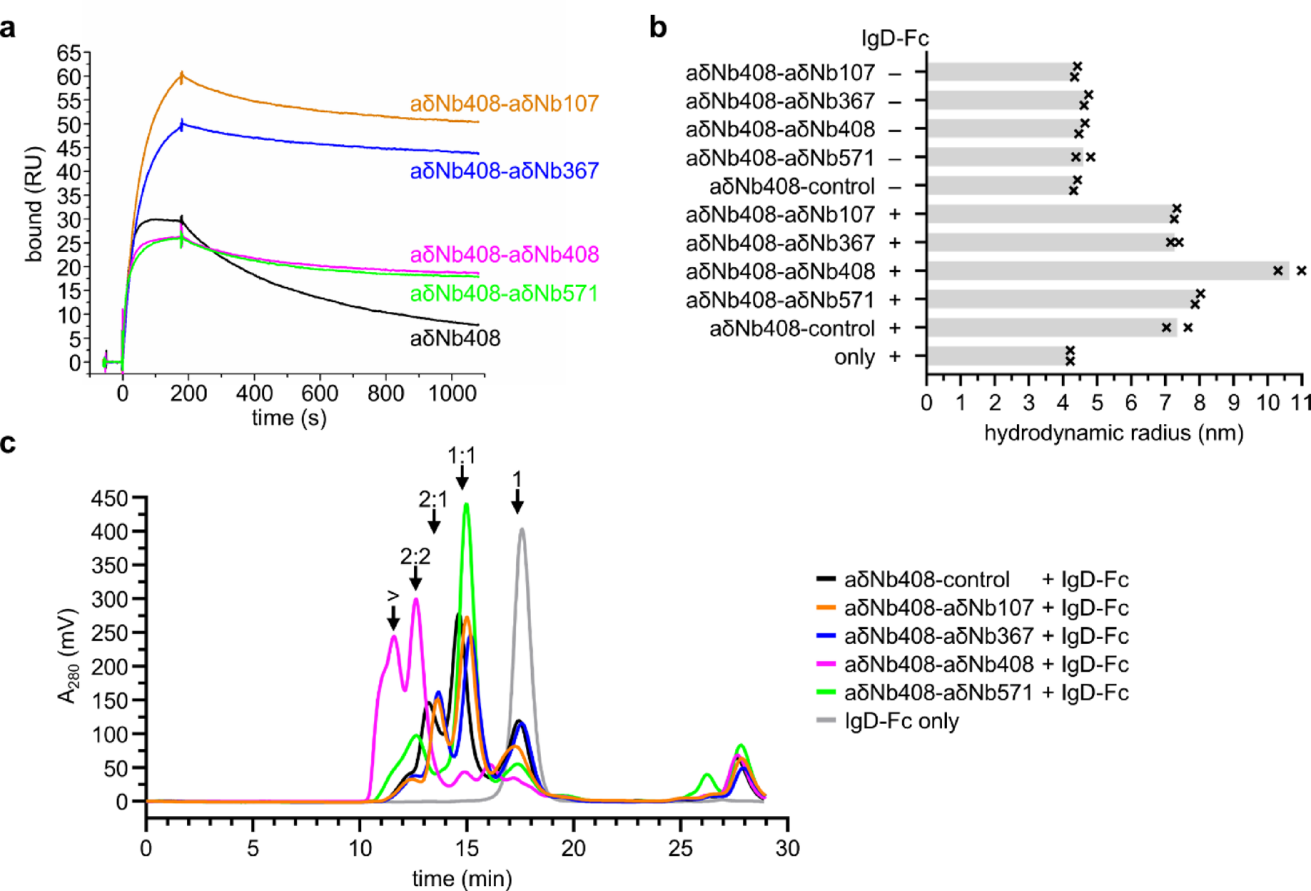
Characterization of anti-IgD Nb pairs. (**a**) Surface plasmon resonance data showing binding of 25 nM aδNb408 or 25 nM aδNb408-Nb pairs to IgD (HAPPID1) captured by its antigen Phl p 7. RU, resonance units. (**b**) Dynamic light scattering of aδNb408-Nb pairs (assembled using DoubleCatcher) pre-incubated with (or without) IgD-Fc. Hydrodynamic radius was plotted, with the mean shown as a gray bar and the individual data points plotted as black crosses (n = 2). (**c**) Size exclusion chromatogram of aδNb408-Nb pairs post-DLS in complex with IgD-Fc, or IgD-Fc only. Approximate locations of inferred interaction stoichiometries are indicated. 1, unbound Nb pairs or unbound IgD-Fc; 1:1, complex formed by one Nb pair interacting with one molecule of IgD-Fc; 2:1, complex formed by two Nb pairs interacting with one molecule of IgD-Fc; 2:2, complex formed by two Nb pairs interacting with two molecules of IgD-Fc; > , complex formed of more than two Nb pairs and two molecules of IgD-Fc.

To perform a more thorough investigation of how the anti-IgD Nb pairs interact with IgD, we chose aδNb408-aδNb107, aδNb408-aδNb367, aδNb408-aδNb408 and aδNb408-aδNb571 for further study. We performed SPR, capturing IgD by its antigen Phl p 7 and testing binding of monovalent aδNb408 or aδNb408 in bivalent and bispecific form (Fig. 5a). All four aδNb408-Nb pairs showed greater than ten-fold slower dissociation from IgD compared with aδNb408 alone, confirming that there are avidity effects at play that stabilize the interaction. It should be noted that the binding of bivalent and bispecific constructs can be dependent on the density and spacing of the target on the surface of the SPR chip, making it difficult to quantify avidity effects. To better understand whether the Nb pairs interact with a single molecule of IgD in an intramolecular manner or cross-link IgD in an intermolecular manner, we performed dynamic light scattering (DLS) experiments (Fig. 5b, Supplementary Fig. S8). All Nb pairs and Nb pairs in complex with IgD-Fc showed monomodal size distributions, with all complexed Nb pairs exhibiting polydispersity. The Nb pairs on their own showed a very similar size distribution compared with each other. However, when they were incubated with IgD-Fc, bivalent aδNb408-aδNb408 showed a much larger hydrodynamic radius than any of the other Nb pairs, suggesting that intermolecular interactions were being formed, with the IgD-Fc cross-linked. To further visualize this, we performed SEC of the samples after DLS analysis (Fig. 5c). aδNb408-aδNb408 started eluting shortly after the void volume of the column, confirming cross-linking of IgD-Fc. aδNb408-aδNb571 showed a main peak, likely a 1:1 interaction between Nb pair and IgD-Fc, as well as a second peak eluting earlier, possibly suggesting some cross-linked IgD-Fc. Nb pairs aδNb408-aδNb107 and aδNb408-aδNb367 mirrored the aδNb408-control, indicating that they formed intramolecular interactions with IgD, but did not lead to formation of higher-order oligomers.

However, changing the linker length or rigidity between the Nbs could potentially influence how they interact with IgD. To test this, we performed Nb assembly with DoubleCatcher H-Lock, which in contrast to the (glycine-serine-glycine)_3_ linker in DoubleCatcher contains a 38 amino acid helical linker^21^, and again performed SEC after incubation with or without IgD-Fc (Supplementary Fig. S9). Using a different Nb assembly platform appeared to have little effect on the interaction between IgD-Fc and the three Nb pairs aδNb408-aδNb107, aδNb408-aδNb367 and aδNb408-aδNb571. However, it changed the extent of oligomerization mediated by aδNb408-aδNb408. While the majority of IgD-Fc still appeared to be in a 2:2 complex or larger with Nb pair aδNb408-aδNb408, an additional 1:1 interaction peak suggested intramolecular interaction was now also observable. This confirmed that the relative orientation and flexibility of the two Nbs could influence interaction selectivity and might be tunable for different applications. Nb pairs with increased avidity and specificity or selectivity can be particularly useful tools in research and diagnostics. The three bispecific Nb pairs that we have characterized here preferentially bound to IgD in an intramolecular manner in solution, while bivalent aδNb408 preferentially cross-linked in an intermolecular manner.

## Discussion

Nbs against human IgE^22^, human IgG^23,24^ and human IgM^25^ have been described, and many other binder formats are available against human isotypes IgA, IgE, IgG and IgM, including affibodies against human IgA^26^. In contrast, anti-IgD reagents have been limited to traditional antibody formats, often of a commercial nature with undisclosed sequence, or in early work derived from hybridomas of unknown sequence, and not easily produced recombinantly. Here we characterized four anti-human IgD Nbs, binding to the Fc region of IgD. Nbs are easily expressed in bacterial expression systems, on a large scale and in a high-throughput manner. The four Nbs displayed distinct binding kinetics and interaction stoichiometries, offering a diverse range of potential applications. Fc regions of immunoglobulins (Ig-Fc) interact with their respective Fc receptors in different ways; for instance, FcγRIII binds to IgG-Fc with 1:1 stoichiometry^27^, while FcRn binds to IgG-Fc with 2:1 stoichiometry^28^. It remains to be determined whether there is only one binding site for aδNb571 on IgD, or whether binding of the first aδNb571 molecule makes a second binding site unavailable. The anti-IgD Nbs described here recognized different epitopes, with both Cδ2 and Cδ3 targeted. During epitope binning on an anti-His chip we observed subtle differences in Nb binding to IgD-Fc for different Nb captures. A marked dissociation of IgD-Fc from aδNb571 was induced by aδNb107, aδNb367 and aδNb408, but not vice versa. However, a much more modest induced dissociation phenomenon was observed when the same experiment was performed with biotinylated aδNb571 immobilized onto an SA chip while testing it as an SPR capture tool. Further studies will be needed to establish a mechanism and rule out proximity effects, but these experiments raise the possibility of allosteric communication within IgD-Fc. Allostery has been observed for other Ig-Fc, such as in IgE-Fc, where it plays an important functional role^29^. The asymmetry of the induced dissociation observed could be due to aδNb571 being more prone to this phenomenon as a 1:1 binder. A single Fc molecule pre-bound to two molecules of aδNb107, aδNb367 or aδNb408 might only release one binding site when aδNb571 subsequently binds, thus being retained on the chip through binding via the second site. Exploring whether there is allostery in IgD-Fc will be helped by having well-defined binders to the IgD-Fc region, such as the Nbs described here. We showed that all four Nbs aδNb107, aδNb367, aδNb408 and aδNb571 were specific to IgD and did not bind to other human isotypes, but we have not yet investigated whether any of these anti-human IgD Nbs cross-react with IgD from other species. Residues in the Cδ2 and Cδ3 domains that contribute to the unique structural features of human IgD-Fc are conserved in a variety of mammalian species^8^. The anti-IgD Nbs described here will likely show cross-reactivity with IgD from nonhuman primates. Mouse and rat IgD do not contain the equivalent of a Cδ2 domain^30,31^, excluding aδNb408 as a possible cross-reactive binder in these two species. Just over 50% of residues within the Cδ3 domain of human and mouse IgD-Fc are identical (Supplementary Fig. S10), and we would not necessarily expect cross-reactivity to mouse IgD, although it is difficult to generalize for different epitopes. IgD from other species, including reptiles and fishes, show extensive differences in number of Cδ domains^32^.

aδNb408 has the required characteristics of an effective purification tool for IgD, as it showed good capture and easy elution at pH 3.5. Small amounts of impurities were observed, and we would recommend adding a size exclusion purification step to obtain highly pure, monomeric IgD, especially when performing purification in the presence of high concentrations of albumin. Engineering of aδNb408 using histidine scanning mutagenesis might allow even gentler elution conditions to be used^33^, avoiding the purification of aggregate. The aδNb408 affinity resin can also be used for co-immunoprecipitation experiments to identify as yet unknown molecular interactors of IgD. aδNb107 and aδNb408 proved to be good capture tools for SPR. It is possible that the poor performance of aδNb367 and aδNb571 in this regard could be improved using a different immobilization approach. Instead of performing direct biotinylation using NHS coupling chemistry, expressing the Nbs with an AviTag and performing biotinylation using BirA^34^ could be a useful alternative. However, some Nbs do not withstand the low pH regeneration conditions needed to remove captured material from a high affinity interaction during SPR experiments.

Using Nbs as bispecific pairs can improve the specificity and selectivity of detection. While we tested cell-binding using the Namalwa B cell line, we would expect the Nbs to also detect IgD on the surface of human primary B cells, where IgD should have similar glycosylation patterns and interact with identical co-receptors. Here we used two versions of the DoubleCatcher platform to assemble Nb pairs, which should result in different spacing of the two Nbs, and we saw subtle differences in intramolecular versus intermolecular binding to IgD as a result. Spacing and linker geometry might also influence avidity effects. This could be further explored using other DoubleCatcher architectures with altered geometries^21^, or completely different approaches to generate bispecifics, including expression as a single fusion protein. When paired with anti-idiotype Nbs, intramolecular cross-linking of the Fab and Fc regions of IgD might permit investigation of the flexibility of the hinge region or as a stabilization approach for structural studies. Intermolecular cross-linking of the IgD B cell antigen receptor by anti-IgD antibodies has an effect on B cell survival^35^, however, whether bivalent aδNb408 has a similar effect on B cells remains to be explored. In summary, we envisage the four anti-human IgD Nbs presented here will be valuable in a range of applications, including detection, imaging, structural studies and more, promoting and facilitating studies of this least well understood class of antibody.

## Methods

### Plasmids and cloning

Cloning was performed using standard PCR methods with Q5 High-Fidelity 2× Master Mix and NEBuilder HiFi DNA Assembly Cloning Kit (NEB). Coding sequences were verified by Sanger sequencing (Source Bioscience or Eurofins Genomics).

The pVITRO1-HAPPID1 construct (Addgene 204626), human anti-Phl p 7 IgD 102.1F10, has been described^16^. A pVITRO1-HAPPID1 construct with a C-terminal glycine_4_-serine linker, SpyTag003^20^, glycine-serine-glycine linker and His_6_-tag on the heavy chain was derived from pVITRO1-HAPPID1.

The pcDNA3.4-IgD-Fc construct with a C-terminal His_6_-tag has been described^8^. A pcDNA3.4-IgD-Fc construct with a C-terminal glycine_4_-serine linker and SpyTag003 was derived from His-tagged pcDNA3.4-IgD-Fc. A pcDNA3.4-Cδ2 (amino acids Ser292 to Ala398) construct with a C-terminal glycine_4_-serine linker and SpyTag003 was derived from SpyTagged pcDNA3.4-IgD-Fc. A pairwise sequence alignment between the human IgD-Fc used in this study (UniProt P01880-1) and mouse IgD-Fc (UniProt P01881) was performed with EMBOSS Needle^36^.

The pET151-Ole e 3 construct with Ala70Cys and C-terminal tryptophan residue was derived analogously to Phl p 7^37^.

Anti-IgD Nbs aδNb107 (Addgene 220319), aδNb367 (Addgene 220320), aδNb408 (Addgene 220321) and aδNb571 (Addgene 220322) in pET-15b were codon-optimized for *E. coli* expression and synthetized by GenScript, with an N-terminal periplasmic leader and a C-terminal TEV cleavage site, followed by a glycine-serine linker and His_6_-tag. SpyTagged Nbs were derived from pET-15b-aδNb107, pET-15b-aδNb367, pET-15b-aδNb408 and pET-15b-aδNb571 for intracellular expression, respectively, with a C-terminal glycine_4_-serine linker, SpyTag003, glycine_2_-serine linker and His_6_-tag. A multiple sequence alignment of the protein sequences was performed with Clustal Omega version 1.2.4^36^. The SpyTagged anti-idiotype control Nb was derived from pET-15b-aδNb063 (Addgene 228464).

The pDEST14-SpySwitch plasmid (Addgene 184225)^38^, pDEST14-SpyCatcher003 S49C plasmid (Addgene 133448)^20^, pDEST14-DoubleCatcher α-Lock plasmid (Addgene 216286)^21^, pDEST14-DoubleCatcher plasmid (Addgene 216284)^21^ and pDEST14-DoubleCatcher H-Lock plasmid (Addgene 216285)^21^ were a gift from Mark Howarth. The pET28a-MBP-super TEV protease plasmid was a gift from Mark Howarth (Addgene 171782) and was expressed and purified in a manner similar to that described previously^39^.

### Catcher expression, resin coupling and fluorescent labeling

SpySwitch, SpyCatcher003 S49C, DoubleCatcher α-Lock, DoubleCatcher and DoubleCatcher H-Lock were expressed in OverExpress C41(DE3) *E. coli* (LGC) by IPTG induction and purified by Ni–NTA affinity chromatography. SpyCatcher003 S49C and SpySwitch were coupled to SulfoLink Coupling Resin as previously described^38^. For AF488-labeling, DoubleCatcher α-Lock and SpyCatcher003 S49C were reduced in labeling buffer (50 mM Tris, 150 mM NaCl, 1 mM TCEP, pH 7.4) for 30 min at room temperature (RT) and incubated with a three-fold (SpyCatcher003 S49C) or 5.5-fold (DoubleCatcher α-Lock) excess of Alexa Fluor 488 C_5_ Maleimide for 4 h rolling at RT. Excess dye was removed by desalting and dialysis. DoubleCatcher α-Lock was labeled with two molecules of AF488 dye via its two cysteine residues, thus abolishing disulfide bond formation, making it near-identical to DoubleCatcher.

### Antibody, antibody fragment and antigen expression and purification

Human anti-Phl p 7 IgD 102.1F10 (HAPPID1) was expressed from stably transfected FreeStyle 293-F cells^16^ growing in spinner flasks using DMEM supplemented with 10% (v/v) FBS, 2 mM L-glutamine, 100 U/mL penicillin, 100 μg/mL streptomycin and 50 µg/mL hygromycin B (referred to hereafter as pVITRO spinner medium) at 37 °C and 75 rpm with 5% (v/v) CO_2_. HAPPID1 was purified by aδNb408 affinity chromatography, as described later. A stably transfected FreeStyle 293-F cell line for SpyTagged HAPPID1 was generated by hygromycin selection and SpyTagged HAPPID1 was expressed as above. His-tagged IgD-Fc for panning was expressed in Expi293F cells using the ExpiFectamine 293 Transfection Kit (both Thermo Fisher) according to the manufacturer’s instructions and purified as described previously^8^. SpyTagged IgD-Fc and Cδ2 were transfected into FreeStyle 293-F cells (Thermo Fisher) and stable cell lines were generated by geneticin selection. Expression of IgD-Fc and Cδ2 was performed in spinner flasks in DMEM supplemented with 10% (v/v) FBS, 2 mM L-glutamine and 500 µg/mL geneticin at 37 °C with 5% (v/v) or 8% (v/v) CO_2_ at 75 rpm. SpyTagged HAPPID1, IgD-Fc and Cδ2 were purified by SpySwitch affinity chromatography^38^ and further purified by SEC on a Superdex 200 Increase (HAPPID1 and IgD-Fc) or Superdex 75 Increase 10/300 GL column (Cδ2). For experiments involving Nb pairs, IgD-Fc was purified by aδNb408 affinity chromatography. Human anti-Phl p 7 IgE 102.1F10 (HAPPIE1) was expressed from a stably transfected HEK293F cell line and purified by anti-IgE affinity chromatography and SEC as described previously^37^. IgA, IgG and IgM from human serum were purchased from Merck for investigating isotype specificity of anti-IgD Nbs. Molecular weights used to calculate molarity were based on monomeric units, with 160 kDa assumed for IgA, 150 kDa assumed for IgG and 180 kDa assumed for IgM.

Ole e 3 was expressed and purified as previously described for Phl p 7^16^.

### Generation of anti-IgD Nbs

Generation of anti-IgD Nbs was outsourced to the VIB Nanobody Core (Vrije Universiteit Brussel) and performed according to their standard protocols, as described previously^16,40,41^. Briefly, an alpaca and a llama were each immunized with six injections of ~ 100 µg IgD (HAPPID1). Anticoagulated blood was collected from each animal four and eight days after the last IgD injection, lymphocytes were isolated and total RNA was extracted. For each animal an independent Nb library was derived using a 1:1 ratio of total RNA from days 4 and 8 post-injection. Each of the libraries was panned on IgD, with the output mixed for a second round of panning. Separately to the panning on IgD, the libraries from the alpaca and the llama were each panned on His-tagged IgD-Fc, and the output mixed for two further rounds of panning. Initial ELISA screening was performed by the VIB Nanobody Core. For further screening purposes performed in-house using SPR, pMECS-aδNbs were expressed in TG1 cells as protein III fusion proteins by IPTG induction and TES extraction was used to prepare periplasmic extracts^40^.

### Nanobody expression

His-tagged Nbs were expressed in BL21 Star(DE3) (Thermo Fisher) or BL21(DE3) *E. coli* (NEB) at 18 °C in ZYP-5052 autoinduction medium^42^ and bacterial pellets frozen at -70°C. Periplasmic extract was prepared by resuspending bacterial pellets in buffer A (10 mM phosphate, 500 mM NaCl, 2.7 mM KCl, 25 mM imidazole, pH 7.4 + 0.1% (w/v) NaN_3_) supplemented with Benzonase, incubating for 45 min rolling at RT, and centrifuging at 2100g or 8900 g for 30 min at 4 °C. Nbs were purified by Ni–NTA Superflow resin using buffer A as wash buffer and eluting in buffer B (PBS + 500 mM imidazole + 0.1% (w/v) NaN_3_, pH 7.4). Nbs were desalted into HBS-az (10 mM HEPES, 150 mM NaCl, pH 7.4 + 0.1% (w/v) NaN_3_) using NAP-10 columns.

To generate Nbs that did not contain a His_6_-tag, TEV cleavage was performed using MBP-super TEV protease at a 1:20 molar ratio at 4 °C overnight. Any remaining His-tagged protein was removed by incubation with HIS-Select Nickel Affinity Gel for 30 min rolling at RT.

SpyTagged Nbs were expressed as above. Lysate was prepared by incubating cells in buffer A + 1% (v/v) IGEPAL CA-630, supplemented with Lysonase and protease inhibitors, for 30 min rolling at RT and centrifuging at 30,000 g for 30 min at 4 °C. Purification from cleared lysate was performed by Ni–NTA affinity chromatography as described above.

To use the Nbs as capture tools in SPR, SpyTagged Nbs were biotinylated with equimolar amounts of EZ-Link NHS-LC-Biotin in PBS pH 7.3 for 2 h on ice, then desalted into HBS-az to remove excess biotinylation reagent.

### Stoichiometry by SEC

To determine the stoichiometry of Nbs to IgD-Fc, complexes were set up with IgD-Fc at 2 µM and incubated overnight at 4 °C. The complexes were analyzed using a Superdex 200 Increase 10/300 GL column in HBS-az at 10 °C. Data were analyzed and plotted in GraphPad Prism version 10.2.2.

### Surface plasmon resonance

SPR experiments were performed using a Biacore T200 instrument at 25 °C with 10 mM HEPES pH 7.4, 150 mM NaCl, 0.05% (v/v) surfactant P20 as running buffer, supplemented with 5 mM CaCl_2_ for experiments involving Ole e 3 or Phl p 7.

To determine the binding affinities of the Nbs to IgD, an anti-His chip was used for ligand capture. An anti-His-tag antibody was immobilized onto a Series S Sensor Chip CM5 using a His Capture Kit (Cytiva), with amine coupling adapted from the manufacturer’s instructions. 100 nM His-tagged Nbs or His-tagged IgD were captured for 180 s at 10 µL/min. A two-fold dilution series of IgD-Fc or TEV-cleaved Nbs was flowed over for 180 s at 20 µL/min, with a dissociation phase of 900 s. Finally, regeneration was performed with 100 mM glycine pH 2.0 for 60 s at 10 µL/min. For epitope binning, 100 nM IgD-Fc was additionally captured for 180 s at 10 µL/min after His-tag capture. Isotype specificity was assessed using the anti-His chip to capture 100 nM His-tagged Nbs for 180 s at 10 µL/min, before flowing over ~ 500 nM IgA, IgE, IgG or IgM for 180 s at 20 µL/min, with a dissociation phase of 600 s. 50 nM IgD was used as a control in the same experiment. Regeneration was performed as above.

To use the anti-IgD Nbs as capture tools for IgD, biotinylated Nbs were immobilized onto a Series S Sensor Chip SA, with at least 1000 resonance units of biotinylated Nbs retained. IgD-Fc or IgD were captured for 180 s at 10 µL/min, before a two-fold dilution series of TEV-cleaved Nbs or Ole e 3 was flowed over for 180 s or 240 s at 20 µL/min, with a dissociation phase of 800 s or 900 s. Regeneration was performed with 100 mM glycine pH 3.5 (for aδNb408) or 100 mM glycine pH 2.5 (for aδNb107, aδNb367 and aδNb571) for 60 s at 10 µL/min.

Preparation of an SA-Phl p 7 chip has been previously described^16^. IgD was captured by its antigen Phl p 7 for 180 s at 10 µL/min. aδNb408 or aδNb408-Nb pairs were flowed over for 180 s at 20 µL/min, with a dissociation phase of 900 s. Regeneration was performed with 100 mM glycine pH 2.5 for 60 s at 10 µL/min.

Experiments were performed in duplicate. Biacore T200 Evaluation software version 1.0 was used to perform double-reference subtraction^43^, unless otherwise indicated. Binding curves were plotted and fit using Origin 7. k_off_ values were derived from dissociation phase data using a monophasic or biphasic exponential decay function, as appropriate for the data. k_obs_ values were fit from association phase data using the equation y = B_eq_ × (1 – exp(–x × k_obs_)), and plotted against concentration to derive k_on_ values from the slope of a linear fit^44^. K_D_ values were determined from the ratio of k_off_/k_on_.

### Making an aδNb408 affinity resin

For resin-coupling, His-tagged aδNb408 was further purified by SEC using a Superdex 75 Increase 10/300 GL column in coupling buffer (200 mM NaHCO_3_ + 500 mM NaCl, pH 8.3) and concentrated to 444 µM. aδNb408 was coupled to NHS-activated Sepharose 4 Fast Flow resin according to the manufacturer’s instructions, at a 1:2 volume ratio for 18 h rolling at 4 °C. Non-reacted groups were blocked with 500 mM ethanolamine + 500 mM NaCl, pH 8.3 for 3 h rolling at 4 °C, and alternating washes with cold 100 mM Tris–HCl pH 8.0 and 100 mM acetate + 500 mM NaCl, pH 4.0 were performed. The resin was washed with and stored in PBS-az (PBS pH 7.3 + 0.1% (w/v) NaN_3_). All buffer pH values are those at RT.

### Testing the aδNb408 affinity resin

For screening of elution conditions, SpyTagged IgD-Fc was reacted with a two-fold molar ratio of AF488-labeled SpyCatcher003 S49C, to make 11.3 µM IgD-Fc:AF488, and incubated for at least 1 h at 4 °C. ~ 25 µL packed aδNb408 affinity resin was incubated with 500 nM IgD-Fc:AF488 in flow buffer (PBS pH 7.3 supplemented with 1% (w/v) BSA and 0.1% (w/v) NaN_3_) for 1 h rolling at 4 °C. Using an AcroPrep Advance 96-well plate with 0.45 μm Supor membrane, the resin was washed 4 × with 10 CV cold PBS-az with centrifugation at 500 g, 30 s, RT. Elution was performed 4 × with 2 CV cold 100 mM glycine pH 2.5, 100 mM glycine pH 3.0, 100 mM glycine pH 3.5, 100 mM sodium acetate pH 4.0, 100 mM sodium acetate pH 4.5 or PBS pH 7.3, incubating each fraction for 2 min, and neutralizing with 0.4 CV 1 M Tris pH 8.6 (at 4 °C) + 0.1% (w/v) NaN_3_. The resin was washed with 10 CV cold PBS-az, before resuspending in PBS-az. Fluorescence was measured in a black 96-well plate using a POLARstar Omega with 485 nm excitation and 530 nm emission in Omega software version 3.00 R3. MARS Data Analysis Software version 3.01 R2 was used to process the data and data were plotted using GraphPad Prism version 10.2.2.

For evaluation of IgD purification, ~ 50 µL packed aδNb408 affinity resin was incubated with 242 nM IgD in flow buffer or 100 nM/200 nM IgD doped into post-purification pVITRO spinner medium for 1 h rolling at 4 °C in Bio-Spin 6 columns. Centrifugation steps were performed at 500 g, 30 s, 4 °C. After the binding step, the resin was washed 4 × with 10 CV cold PBS-az. Elution was performed 4 × with 2 CV 100 mM glycine + 0.1% (w/v) NaN_3_, pH 2.5 or pH 3.5, incubating each fraction for 2 min, and neutralizing with 0.4 CV 1 M Tris pH 8.6 (at 4 °C) + 0.1% (w/v) NaN_3_. Elution samples were analyzed by SEC on a Superdex 200 Increase 10/300 GL column in PBS-az or HBS-az at 10 °C. Data were plotted using GraphPad Prism version 10.2.2.

### Purification using an aδNb408 affinity column

For large-scale purification of IgD or IgD-Fc from cell culture supernatant, a C 10/10 column was packed with 3 mL aδNb408 affinity resin. Purification was performed at 4 °C using an ÄKTAprime plus at 0.5 mL/min. The resin was equilibrated with 5 CV PBS-az, the cell culture supernatant was passed over, and the resin washed with 20 CV PBS-az. Elution was performed with 30 CV 100 mM glycine + 0.1% (w/v) NaN_3_, pH 3.5 in 1 CV fractions, each neutralized with 0.1 CV 1 M Tris pH 8.6 (at 4 °C) + 0.1% (w/v) NaN_3_. To remove any aggregate and for buffer exchange, a size exclusion polishing step was performed using a Superdex 200 Increase 10/300 GL column.

### Bispecific Nb generation

Bispecific Nb pairs were generated using the DoubleCatcher platform^21^. AF488-labeled DoubleCatcher α-Lock, DoubleCatcher or DoubleCatcher H-Lock were incubated with a 1.25-fold molar excess of SpyTagged Nb 1 for 2 h at 25 °C. A five-fold molar excess of SpyTagged Nb 2, along with MBP-super TEV protease at a 1/12 molar ratio were added and incubated for 6 h at 25 °C. After addition of SpyCatcher003 resin, samples were incubated overnight at 4 °C to remove unreacted Nbs. For DLS analysis, SpyCatcher003 resin was removed using an AcroPrep Advance 96-well plate with 0.45 μm Supor membrane. To generate AF488-labeled monovalent Nbs, SpyTagged Nbs were incubated with equimolar amounts of AF488-labeled SpyCatcher003 at 4 °C overnight. An anti-idiotype Nb to HAPPID1 was used as a control, which is not specific to IgD or IgD-Fc.

### Flow cytometry

The human B cell line Namalwa (ATCC) was cultured in RPMI 1640 medium, supplemented with 10% (v/v) FBS, 2 mM L-glutamine, 100 U/mL penicillin, and 100 μg/mL streptomycin under humidified conditions at 37 °C and 5% (v/v) CO_2_.

2.5 × 10^5^ Namalwa cells were stained with LIVE/DEAD Fixable Near-IR Dead Cell Stain in PBS pH 7.3 at 1/10 of the manufacturer’s instructions. Cells were incubated with 1 µM AF488-labeled Nb:Catcher fusion in flow buffer (PBS pH 7.3 supplemented with 1% (w/v) BSA and 0.1% (w/v) NaN_3_) for 1 h at 4 °C. 1 µM, 500 nM and 250 nM AF488-labeled Nb:Catcher fusion was used for titration. After one wash in flow buffer, cells were acquired on a Cytoflex LX instrument running CytExpert version 2.4.0.28. Data were analyzed and plotted in FlowJo version 10.8.1, with median fluorescence intensity (MFI) calculated. MFI values were normalized using Microsoft Excel and plotted in GraphPad Prism version 10.2.2, with the mean shown as bars.

### Dynamic light scattering

Bispecific or bivalent Nb pairs were incubated with or without IgD-Fc at 10.5 µM in HBS-az (using a 1:1 molar ratio) overnight at 4 °C to allow complex formation. Measurements were performed using a DynaPro Plate Reader III with DYNAMICS software version 8.1.2.144 at 25 °C. Ten acquisitions, each with a 10 s acquisition time, were used to determine the hydrodynamic radius from the regularization fit of the data, using PBS as solvent approximation. The estimated hydrodynamic radii from two independent experiments are presented. Following DLS, samples were diluted to ~ 6.2 µM and analyzed by SEC using a Superdex 200 Increase 10/300 GL column in HBS-az at 10 °C. Data were visualized by plotting in GraphPad Prism version 10.2.2.

## Electronic supplementary material

Below is the link to the electronic supplementary material.

Supplementary Material 1

## Data Availability

The pET-15b-aδNb107, pET-15b-aδNb367, pET-15b-aδNb408 and pET-15b-aδNb571 constructs have been deposited in the Addgene repository (https://www.addgene.org/James_McDonnell/). The data generated and analyzed in the current study are available from the corresponding author on reasonable request.

## References

[1] Lu, L. L., Suscovich, T. J., Fortune, S. M. & Alter, G. Beyond binding: Antibody effector functions in infectious diseases. *Nat. Rev. Immunol.* **18**, 46–61 (2018). 29063907 10.1038/nri.2017.106 PMC6369690

[2] Gutzeit, C., Chen, K. & Cerutti, A. The enigmatic function of IgD: Some answers at last. *Eur. J. Immunol.* **48**, 1101–1113 (2018). 29733429 10.1002/eji.201646547 PMC6033660

[3] Rowe, D. S. & Fahey, J. L. A new class of human immunoglobulins. II. Normal serum IgD. *J. Exp. Med.* **121**, 185–199 (1965). 14253483 10.1084/jem.121.1.185 PMC2137970

[4] Chen, K. & Cerutti, A. New insights into the enigma of immunoglobulin D. *Immunol. Rev.* **237**, 160–179 (2010). 20727035 10.1111/j.1600-065X.2010.00929.x PMC3048779

[5] Shan, M. et al. Secreted IgD amplifies humoral T helper 2 cell responses by binding basophils via galectin-9 and CD44. *Immunity* **49**, 709-724.e8 (2018). 30291028 10.1016/j.immuni.2018.08.013 PMC6366614

[6] Sun, Z. et al. Semi-extended solution structure of human myeloma immunoglobulin D determined by constrained X-ray scattering. *J. Mol. Biol.* **353**, 155–173 (2005). 16157351 10.1016/j.jmb.2005.07.072

[7] Davies, A. M. et al. Crystal structures of the human IgD Fab reveal insights into CH1 domain diversity. *Mol. Immunol.* **159**, 28–37 (2023). 37267832 10.1016/j.molimm.2023.05.006 PMC7614686

[8] Davies, A. M. et al. The crystal structure of human IgD-Fc reveals unexpected differences with other antibody isotypes. *Proteins Struct. Funct. Bioinf.* **93**, 786–800 (2025). 10.1002/prot.26771 PMC11878202 39582378

[9] Marches, R. & Ghetie, V. Interaction between human IgD and *Ricinus *agglutinin. *Scand. J. Immunol.* **24**, 45–48 (1986). 3726458 10.1111/j.1365-3083.1986.tb02068.x

[10] Zehr, B. D. & Litwin, S. D. Human IgD and IgA1 compete for D-galactose-related binding sites on the lectin jacalin. *Scand. J. Immunol.* **26**, 229–236 (1987). 3659839 10.1111/j.1365-3083.1987.tb02256.x

[11] Samuelsson, M. et al. The IgD CH1 region contains the binding site for the human respiratory pathogen *Moraxella catarrhalis* IgD-binding protein MID. *Eur. J. Immunol.* **36**, 2525–2534 (2006). 16906531 10.1002/eji.200535594

[12] Samuelsson, M., Forsgren, A. & Riesbeck, K. Purification of IgD from human serum—A novel application of recombinant *M. catarrhalis* IgD-binding protein (MID). *J. Immunol. Methods* **317**, 31–37 (2006). 17056056 10.1016/j.jim.2006.09.007

[13] Hamers-Casterman, C. et al. Naturally occurring antibodies devoid of light chains. *Nature* **363**, 446–448 (1993). 8502296 10.1038/363446a0

[14] Ghahroudi, M. A., Desmyter, A., Wyns, L., Hamers, R. & Muyldermans, S. Selection and identification of single domain antibody fragments from camel heavy-chain antibodies. *FEBS Lett.* **414**, 521–526 (1997). 9323027 10.1016/s0014-5793(97)01062-4

[15] De Meyer, T., Muyldermans, S. & Depicker, A. Nanobody-based products as research and diagnostic tools. *Trends Biotechnol.* **32**, 263–270 (2014). 24698358 10.1016/j.tibtech.2014.03.001

[16] Vester, S. K. et al. Expanding the anti-Phl p 7 antibody toolkit: An anti-idiotype nanobody inhibitor. *Antibodies* **12**, 75 (2023). 37987253 10.3390/antib12040075 PMC10660547

[17] Zimmermann, I. et al. Generation of synthetic nanobodies against delicate proteins. *Nat. Protoc.* **15**, 1707–1741 (2020). 32269381 10.1038/s41596-020-0304-x PMC7617899

[18] Drake, A. W. et al. Biacore surface matrix effects on the binding kinetics and affinity of an antigen/antibody complex. *Anal. Biochem.* **429**, 58–69 (2012). 22766435 10.1016/j.ab.2012.06.024

[19] Dussault, N. et al. Immunomodulation of human B cells following treatment with intravenous immunoglobulins involves increased phosphorylation of extracellular signal-regulated kinases 1 and 2. *Int. Immunol.* **20**, 1369–1379 (2008). 18689724 10.1093/intimm/dxn090

[20] Keeble, A. H. et al. Approaching infinite affinity through engineering of peptide-protein interaction. *Proc. Natl. Acad. Sci. U. S. A.* **116**, 26523–26533 (2019). 31822621 10.1073/pnas.1909653116 PMC6936558

[21] Driscoll, C. L., Keeble, A. H. & Howarth, M. R. SpyMask enables combinatorial assembly of bispecific binders. *Nat. Commun.* **15**, 2403 (2024). 38493197 10.1038/s41467-024-46599-9 PMC10944524

[22] Jabs, F. et al. Trapping IgE in a closed conformation by mimicking CD23 binding prevents and disrupts FcϵRI interaction. *Nat. Commun.* **9**, 7 (2018). 29295972 10.1038/s41467-017-02312-7 PMC5750235

[23] Kazemi-Lomedasht, F. et al. Selection and characterization of specific nanobody against human immunoglobulin G. *Monoclon. Antib. Immunodiagn. Immunother.* **34**, 201–205 (2015). 26090598 10.1089/mab.2014.0086

[24] Zhu, D. et al. Novel application of anti-human Fc nanobody for screening high-producing CHO cells for monoclonal antibody. *Eng. Life Sci.* **22**, 608–618 (2022). 36247827 10.1002/elsc.202200028 PMC9550735

[25] Scarrone, M. et al. Development of anti-human IgM nanobodies as universal reagents for general immunodiagnostics. *N. Biotechnol.* **64**, 9–16 (2021). 33984500 10.1016/j.nbt.2021.05.002

[26] Rönnmark, J., Grönlund, H., Uhlén, M. & Nygren, P.-Å. Human immunoglobulin A (IgA)-specific ligands from combinatorial engineering of protein A. *Eur. J. Biochem.* **269**, 2647–2655 (2002). 12047372 10.1046/j.1432-1033.2002.02926.x

[27] Sondermann, P., Huber, R., Oosthuizen, V. & Jacob, U. The 3.2-Å crystal structure of the human IgG1 Fc fragment-FcγRIII complex. *Nature* **406**, 267–273 (2000). 10917521 10.1038/35018508

[28] Sánchez, L. M., Penny, D. M. & Bjorkman, P. J. Stoichiometry of the interaction between the major histocompatibility complex-related Fc receptor and its Fc ligand. *Biochemistry* **38**, 9471–9476 (1999). 10413524 10.1021/bi9907330

[29] Dhaliwal, B. et al. Crystal structure of IgE bound to its B-cell receptor CD23 reveals a mechanism of reciprocal allosteric inhibition with high affinity receptor FcεRI. *Proc. Natl. Acad. Sci. U. S. A.* **109**, 12686–12691 (2012). 22802656 10.1073/pnas.1207278109 PMC3412039

[30] Tucker, P. W., Liu, C.-P., Mushinski, J. F. & Blattner, F. R. Mouse immunoglobulin D: Messenger RNA and genomic DNA sequences. *Science* **209**, 1353–1360 (1980). 6968091 10.1126/science.6968091

[31] Sire, J., Auffray, C. & Jordan, B. R. Rat immunoglobulin delta heavy chain gene: Nucleotide sequence derived from cloned cDNA. *Gene* **20**, 377–386 (1982). 6819978 10.1016/0378-1119(82)90206-2

[32] Chen, K. & Cerutti, A. The function and regulation of immunoglobulin D. *Curr. Opin. Immunol.* **23**, 345–352 (2011). 21353515 10.1016/j.coi.2011.01.006 PMC3109135

[33] Laughlin, T. M. & Horn, J. R. Engineering pH-sensitive single domain antibodies. In *Single-Domain Antibodies. Methods in Molecular Biology* Vol. 2446 (eds Hussack, G. & Henry, K. A.) 269–298 (Humana, 2022). 10.1007/978-1-0716-2075-5_13 PMC9924069 35157278

[34] Fairhead, M. & Howarth, M. Site-specific biotinylation of purified proteins using BirA. In *Site-Specific Protein Labeling. Methods in Molecular Biology* Vol. 1266 (eds Gautier, A. & Hinner, M.J.) 171–184 (Humana Press, 2015). 10.1007/978-1-4939-2272-7_12 PMC4304673 25560075

[35] Yasuda, S. et al. Opposing roles of IgM and IgD in BCR-induced B-cell survival. *Genes Cells* **23**, 868–879 (2018). 30092613 10.1111/gtc.12635

[36] Madeira, F. et al. The EMBL-EBI job dispatcher sequence analysis tools framework in 2024. *Nucleic Acids Res.* **52**, W521–W525 (2024). 38597606 10.1093/nar/gkae241 PMC11223882

[37] Bucaite, G. et al. Interplay between affinity and valency in effector cell degranulation: A model system with polcalcin allergens and human patient-derived IgE antibodies. *J. Immunol.* **203**, 1693–1700 (2019). 31462504 10.4049/jimmunol.1900509 PMC6887533

[38] Vester, S. K. et al. SpySwitch enables pH- or heat-responsive capture and release for plug-and-display nanoassembly. *Nat. Commun.* **13**, 3714 (2022). 35764623 10.1038/s41467-022-31193-8 PMC9240080

[39] Keeble, A. H. et al. DogCatcher allows loop-friendly protein-protein ligation. *Cell Chem. Biol.* **29**, 339-350.e10 (2022). 34324879 10.1016/j.chembiol.2021.07.005 PMC8878318

[40] Pardon, E. et al. A general protocol for the generation of nanobodies for structural biology. *Nat. Protoc.* **9**, 674–693 (2014). 24577359 10.1038/nprot.2014.039 PMC4297639

[41] Schoonaert, L. et al. Identification and characterization of nanobodies targeting the EphA4 receptor. *J. Biol. Chem.* **292**, 11452–11465 (2017). 28526745 10.1074/jbc.M116.774141 PMC5500810

[42] Studier, F. W. Protein production by auto-induction in high-density shaking cultures. *Protein Expr. Purif.* **41**, 207–234 (2005). 15915565 10.1016/j.pep.2005.01.016

[43] Myszka, D. G. Improving biosensor analysis. *J. Mol. Recognit.* **12**, 279–284 (1999). 10556875 10.1002/(SICI)1099-1352(199909/10)12:5<279::AID-JMR473>3.0.CO;2-3

[44] Hulme, E. C. & Trevethick, M. A. Ligand binding assays at equilibrium: Validation and interpretation. *Br. J. Pharmacol.* **161**, 1219–1237 (2010). 20132208 10.1111/j.1476-5381.2009.00604.x PMC3000649

