# Supplementary material for: Anti-IgD nanobodies as novel tools for studying human IgD biology
Source: Sci Rep. 2025 Jul 8;15:24455. doi: 10.1038/s41598-025-09118-4 (PMC12238353; doi:10.1038/s41598-025-09118-4)
Supplement: Supplementary file 1 — Supplementary Material 1 [file 41598_2025_9118_MOESM1_ESM.docx]

**Supplementary Information**

**Anti-IgD nanobodies as novel tools for studying human IgD biology**

Susan K. Vester^1^, Rebecca L. Beavil^1,2^, Alexander Alexandrovich^1,2^, Hannah J. Gould^1^, Andrew J. Beavil^1^, Brian J. Sutton^1^, and James M. McDonnell^1,*^

^1^Randall Centre for Cell and Molecular Biophysics, King’s College London, New Hunt’s House, London, SE1 1UL, United Kingdom

^2^Current address: 272BIO, Kent Science Park, Building 400, The Ventures Building, Sittingbourne, ME9 8AG, United Kingdom

*Corresponding author ([james.mcdonnell@kcl.ac.uk](mailto:james.mcdonnell@kcl.ac.uk))


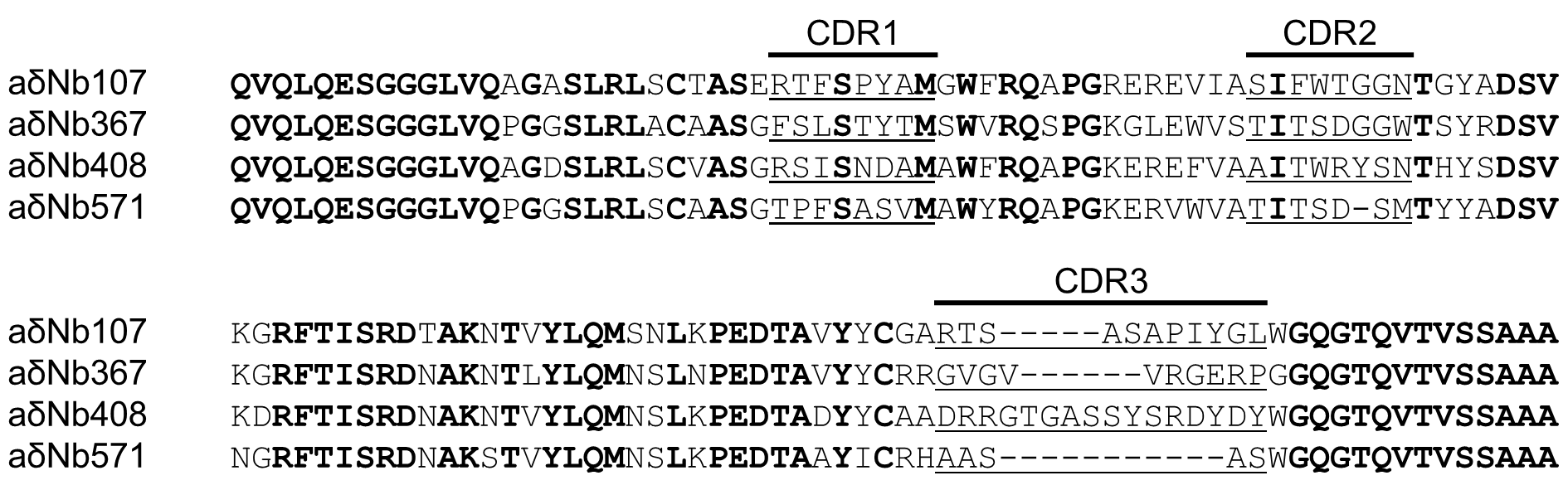


**Supplementary Figure S1: Sequence alignment of anti-IgD Nbs.** Identical amino acids are shown in bold. Approximate locations of complementarity-determining regions (CDRs) are annotated with an underscore.


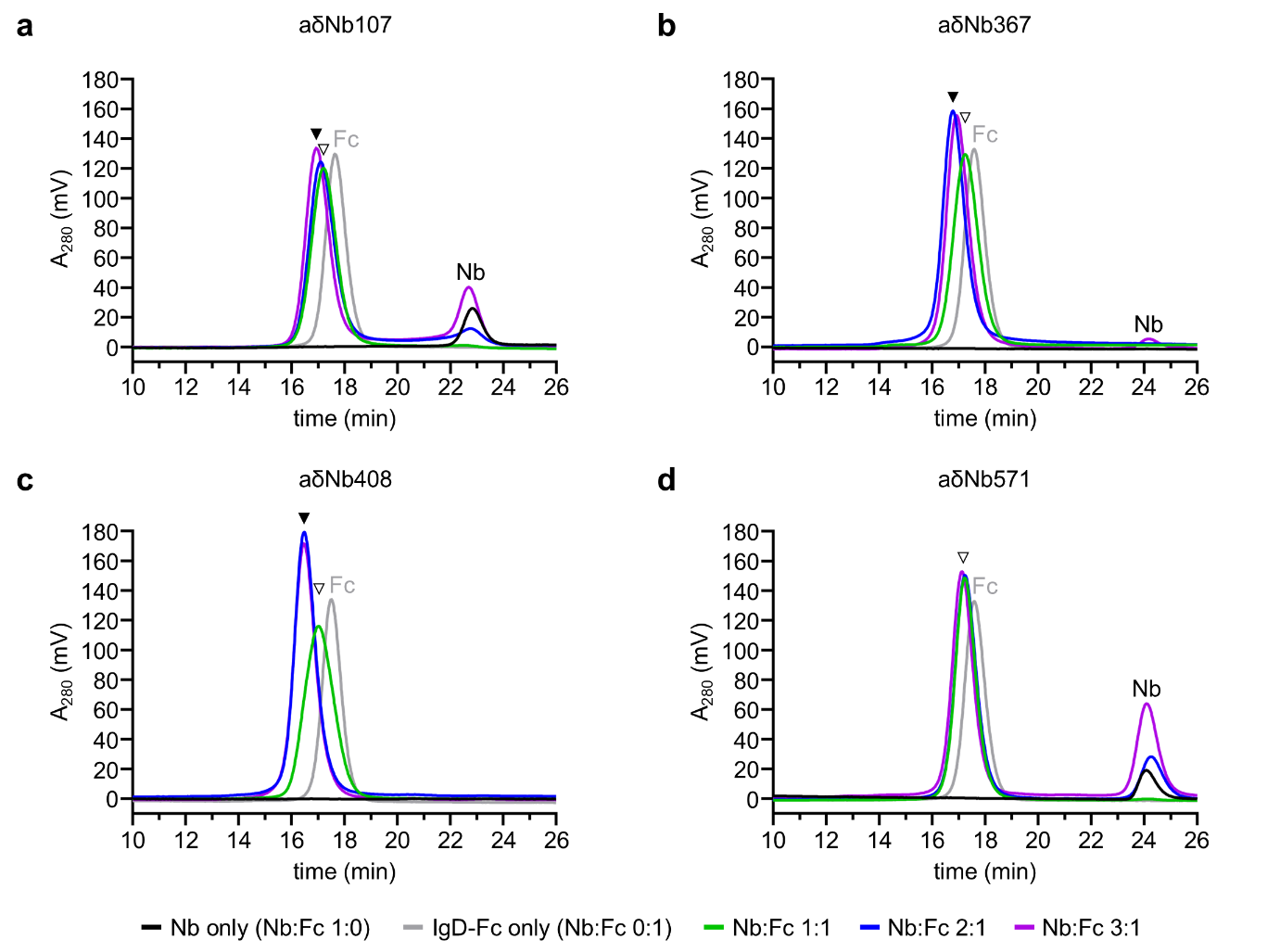


**Supplementary Figure S2: Stoichiometries of anti-IgD Nbs.** Size exclusion chromatograms showing (**a**) aδNb107, (**b**) aδNb367, (**c**) aδNb408 and (**d**) aδNb571 in complex with IgD-Fc. Complexes were set up at 1:1, 2:1 and 3:1 molar ratio to assess the stoichiometries of the Nb interactions with IgD. Empty triangles indicate the location of the 1:1 Nb:Fc complex, filled triangles indicate the location of the 2:1 Nb:Fc complex.

**
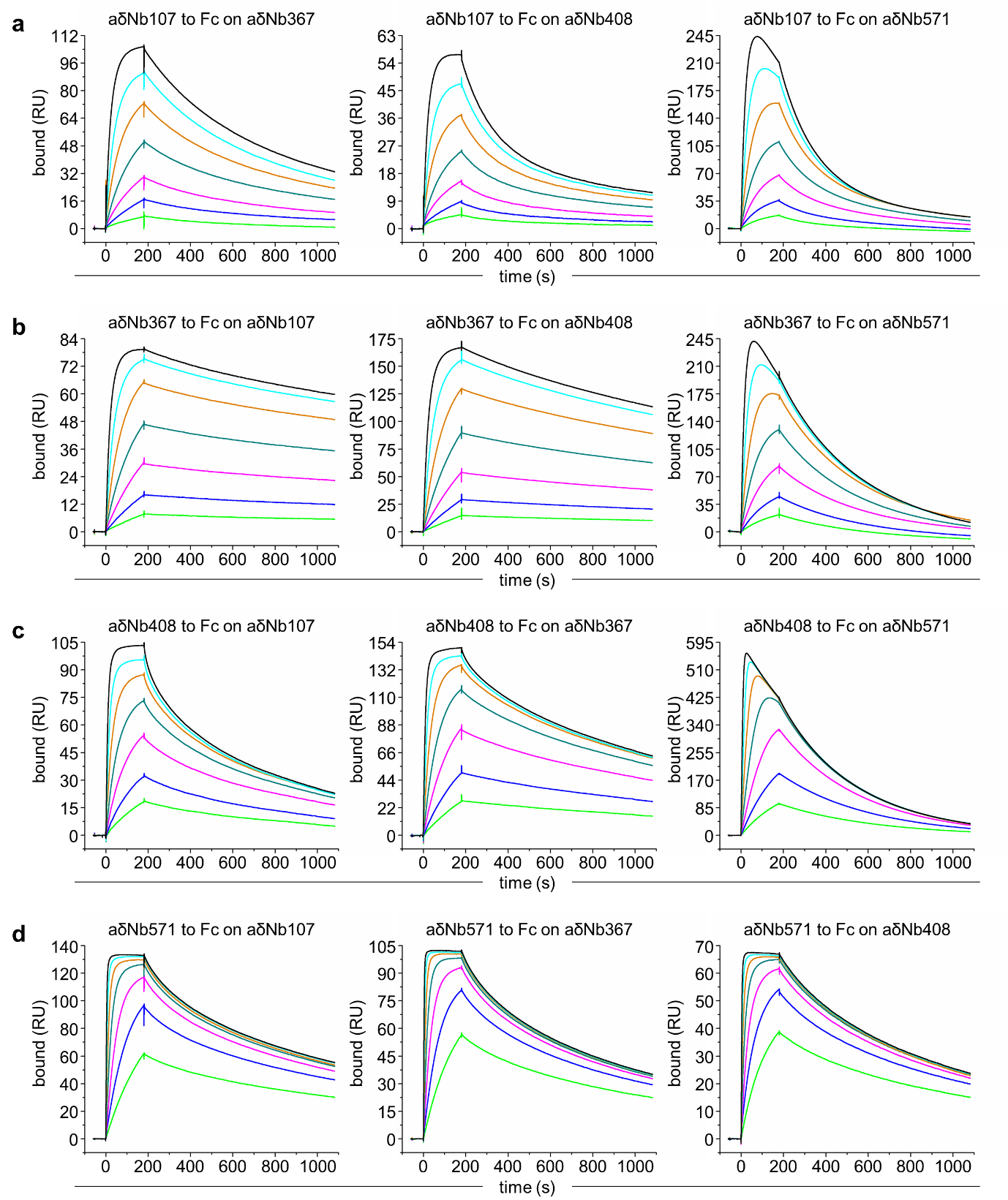
**

**Supplementary Figure S3:** **Epitope binning of anti-IgD Nbs.** His-tagged Nbs were captured by anti-His, IgD-Fc was captured on the Nbs, and two-fold dilution series of TEV-cleaved (**a**) aδNb107, (**b**) aδNb367, (**c**) aδNb408 or (**d**) aδNb571 were flowed over, with the highest concentration 200 nM (black line) and the lowest concentration 3 nM (green line). RU, resonance units.

**
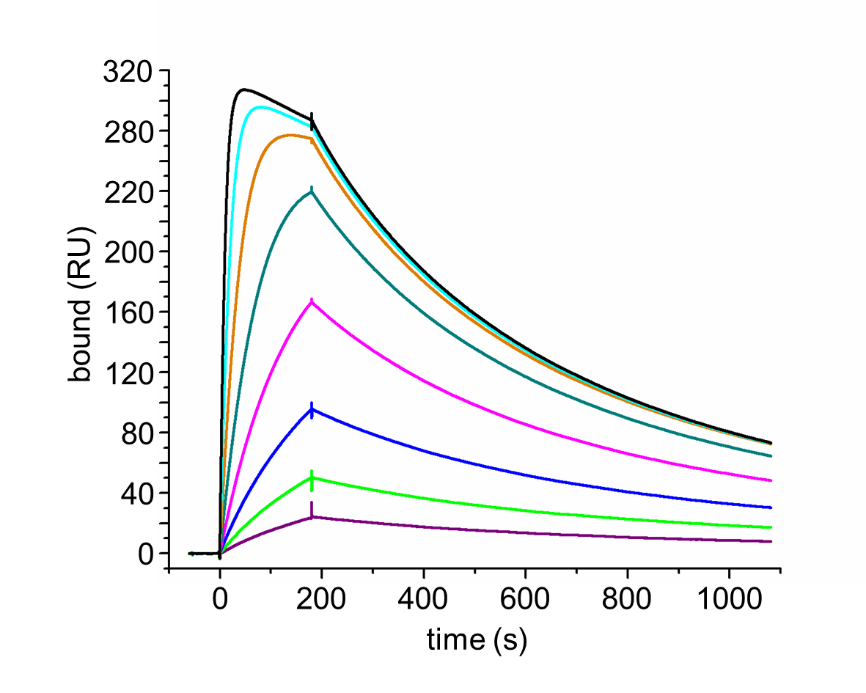
**

**Supplementary Figure S4:** **aδNb408 is a Cδ2 binder.** aδNb408 was captured by anti-His and a two-fold dilution series of Cδ2 was flowed over, with the highest concentration 200 nM (black line) and the lowest concentration 1.6 nM (purple line). RU, resonance units.


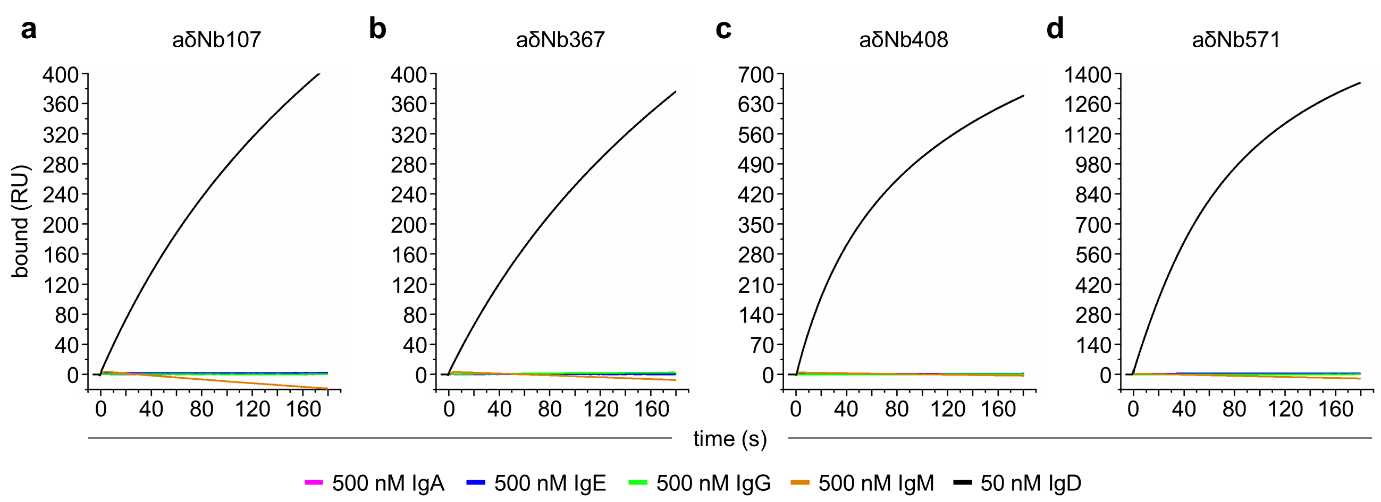


**Supplementary Figure S5: Isotype screening of anti-IgD Nbs.** An anti-His chip was used to capture (**a**) aδNb107, (**b**) aδNb367, (**c**) aδNb408 or (**d**) aδNb571. IgA, IgE, IgG and IgM were flowed over at 500 nM, with IgD used as a positive control at 50 nM. Only the association phase is shown. RU, resonance units.

**
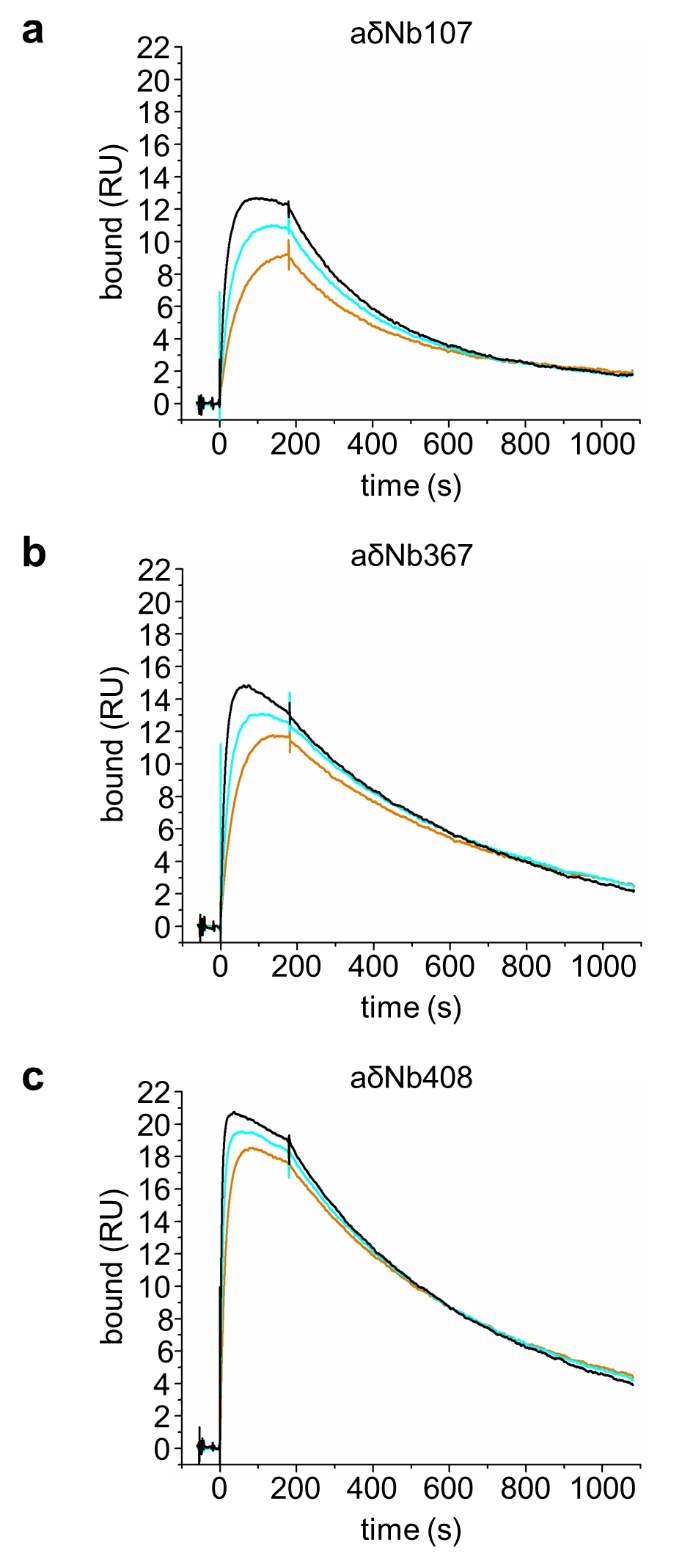
**

**Supplementary Figure S6: aδNb571 is not a suitable capture tool for SPR.** Biotinylated aδNb571 was immobilized onto an SA chip. IgD-Fc was captured by aδNb571 and a two-fold dilution series of (**a**) aδNb107, (**b**) aδNb367 or (**c**) aδNb408 was flowed over, with the highest concentration 200 nM (black line) and the lowest concentration 50 nM (orange line). Due to low capture levels of IgD-Fc, little analyte binding is observed. RU, resonance units.

**
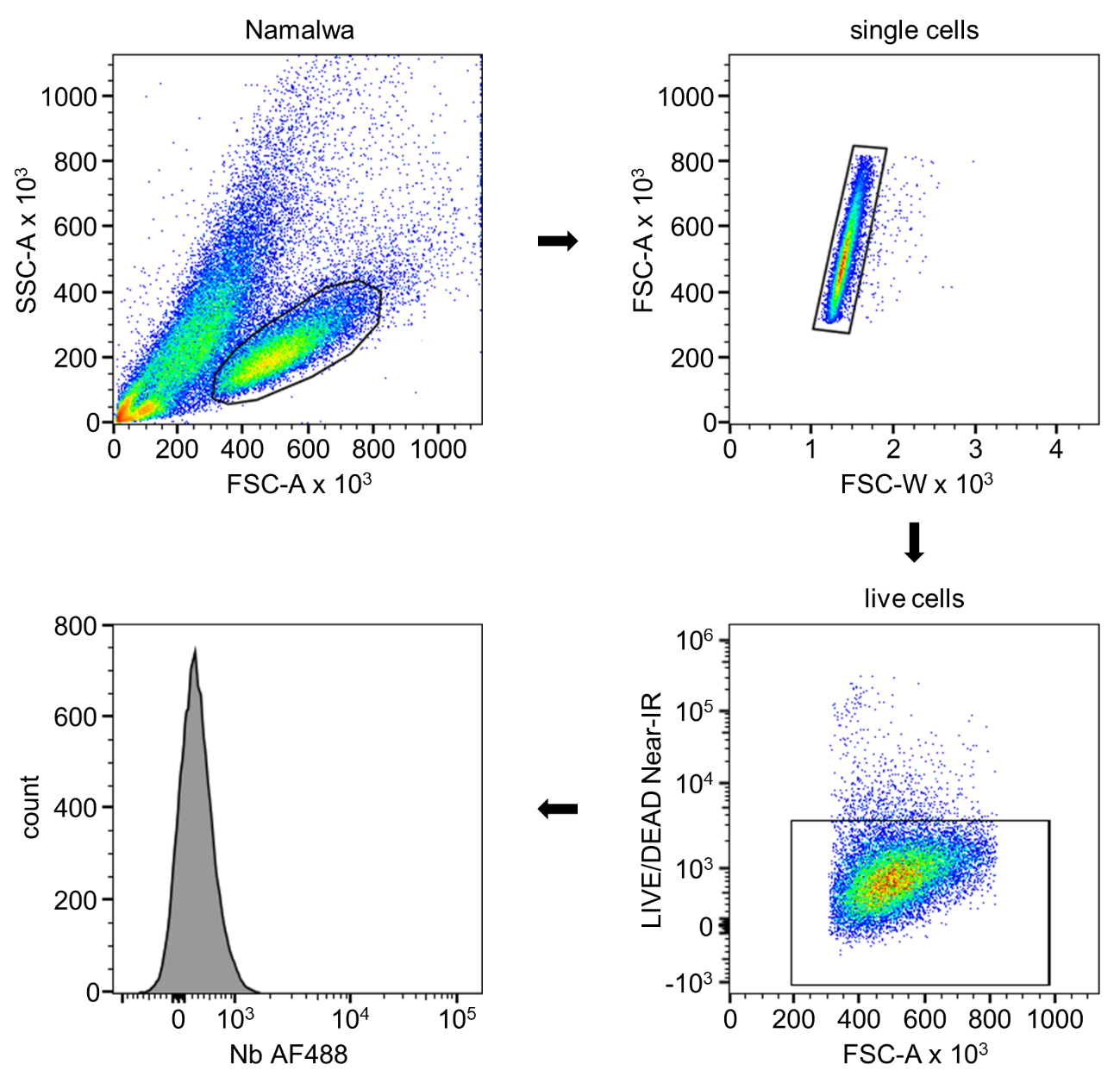
**

**Supplementary Figure S7: Flow cytometry gating strategy.** The main Namalwa cell population was gated for single cells, then live cells, and then histograms (or MFI values) for AF488 fluorescence were presented. Gating shown for untreated cells as a representative example.


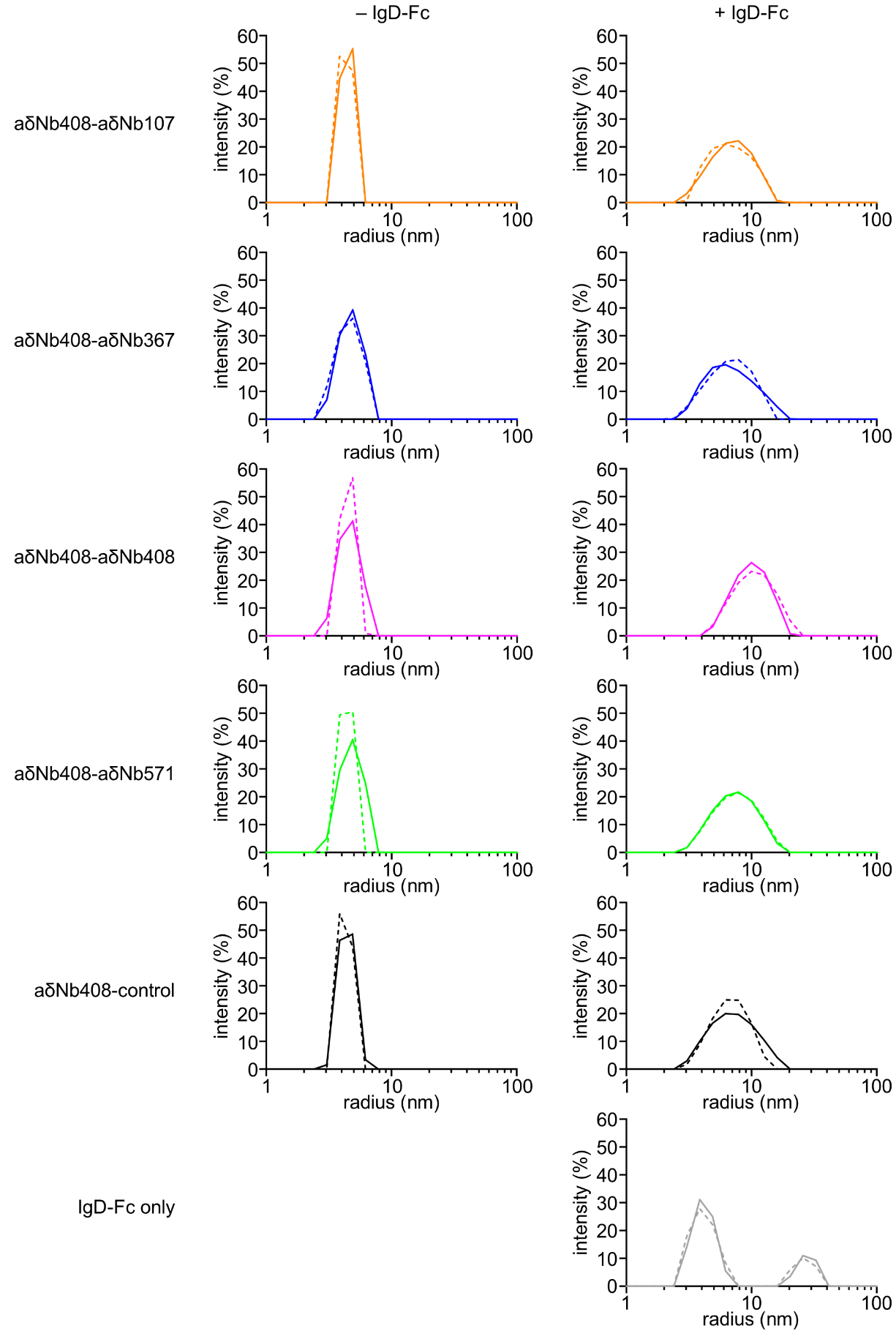


**Supplementary Figure S8: Dynamic light scattering of anti-IgD Nb pairs.** aδNb408-Nb pairs, assembled using DoubleCatcher, were pre-incubated with (right panels) or without IgD-Fc (left panels). Hydrodynamic radius was plotted for duplicate experiments (solid and dashed lines); each experiment consisted of ten technical replicates. The second peak in the IgD-Fc only sample, averaging ~27 nm, makes up only 0.1% of the mass (using the Rayleigh Spheres model) and was not included in the analysis in Figure 5b.


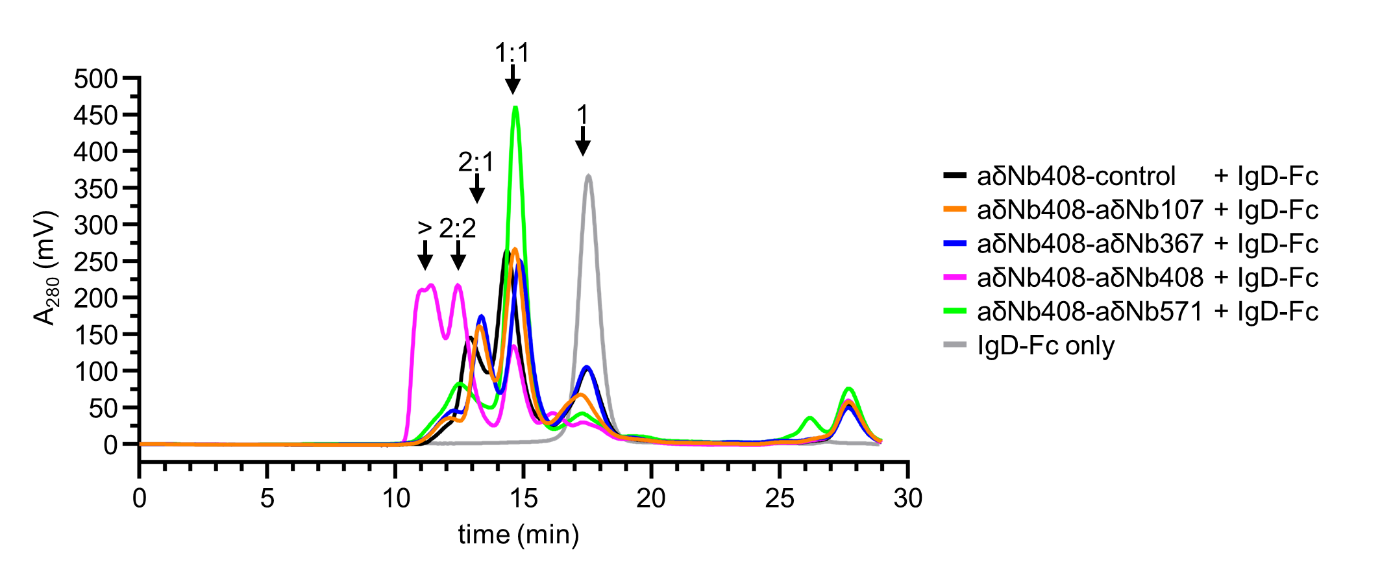


**Supplementary Figure S9:** **Characterization of anti-IgD Nb pairs.** Size exclusion chromatogram of aδNb408-Nb pairs assembled using DoubleCatcher H-Lock in complex with IgD-Fc, or IgD-Fc only. Approximate locations of inferred interaction stoichiometries are indicated. 1, unbound Nb pairs or unbound IgD-Fc; 1:1, complex formed by one Nb pair interacting with one molecule of IgD-Fc; 2:1, complex formed by two Nb pairs interacting with one molecule of IgD-Fc; 2:2, complex formed by two Nb pairs interacting with two molecules of IgD-Fc; >, complex formed of more than two Nb pairs and two molecules of IgD-Fc.


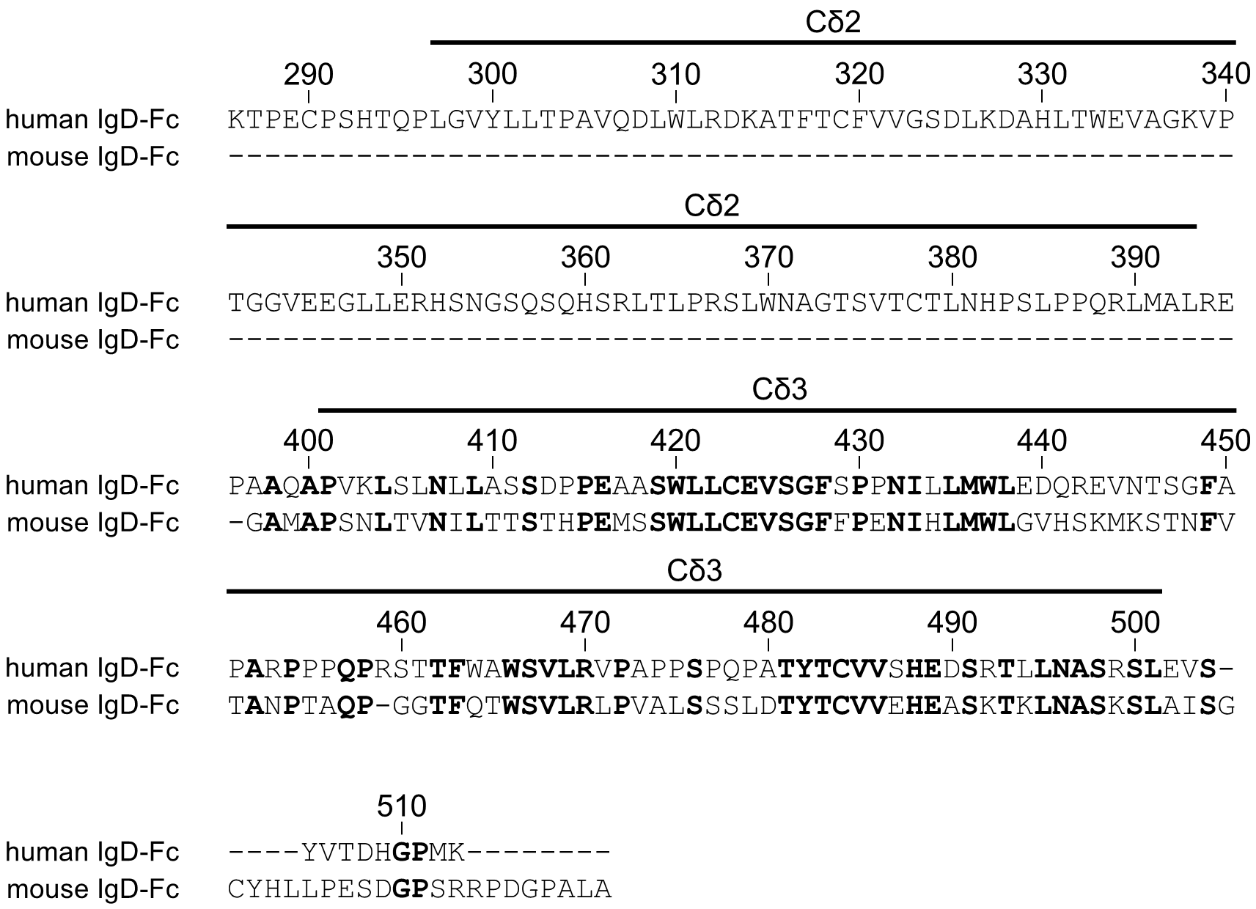


**Supplementary Figure S10: Sequence alignment of the Fc region of human and mouse secreted IgD.** The human IgD-Fc sequence used in this study (UniProt P01880-1) was aligned with the equivalent region from mouse IgD-Fc (UniProt P01881), with the numbering according to human IgD-Fc. Human Cδ2 and Cδ3 domains have been annotated; mouse IgD does not contain the equivalent of a human Cδ2 domain. Identical amino acids are shown in bold.
